# Supplementary figures and images for: Conservation of peripheral nervous system formation mechanisms in divergent ascidian embryos
Source: eLife. 2020 Nov 16;9:e59157. doi: 10.7554/eLife.59157 (PMC7710358; doi:10.7554/eLife.59157)

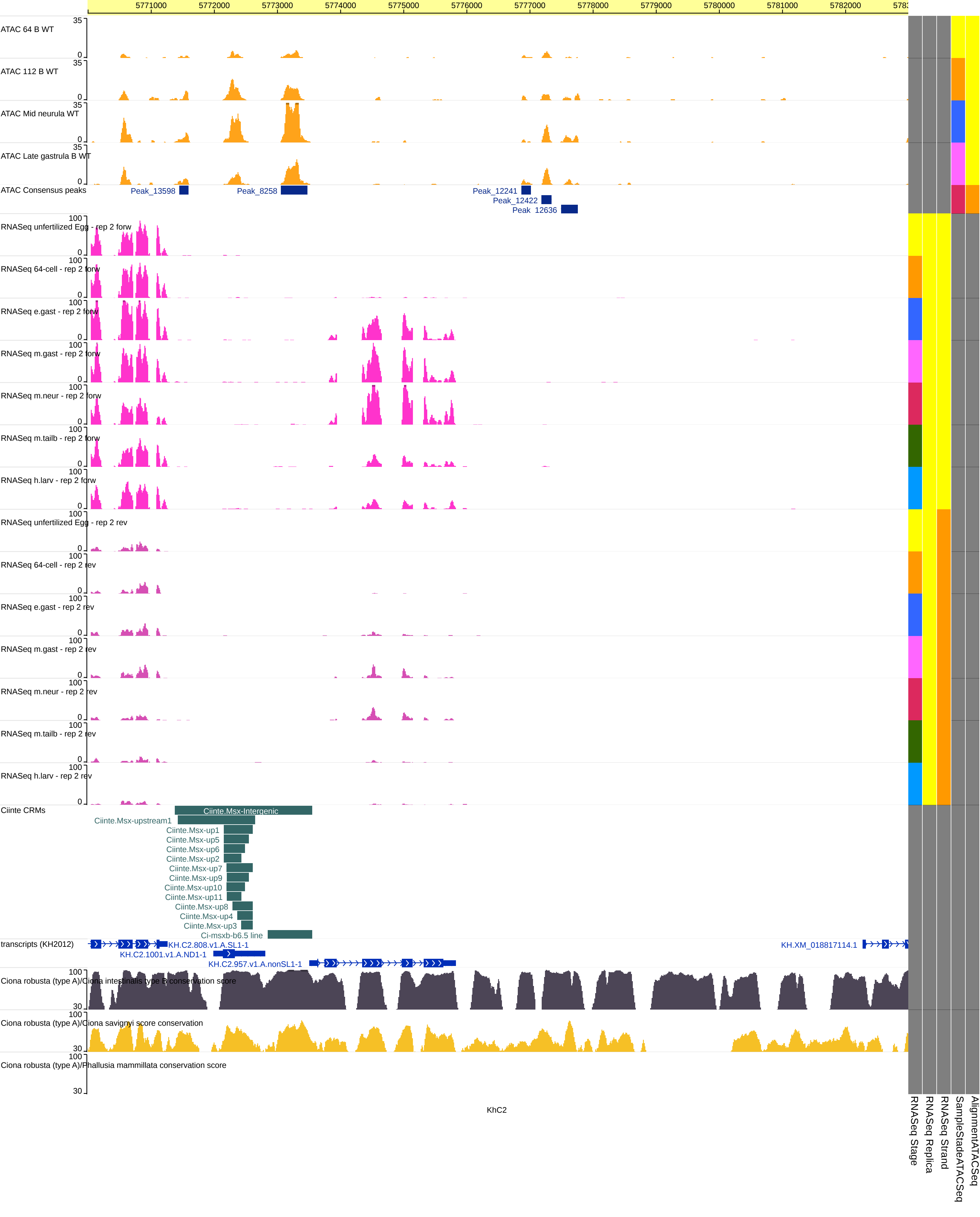

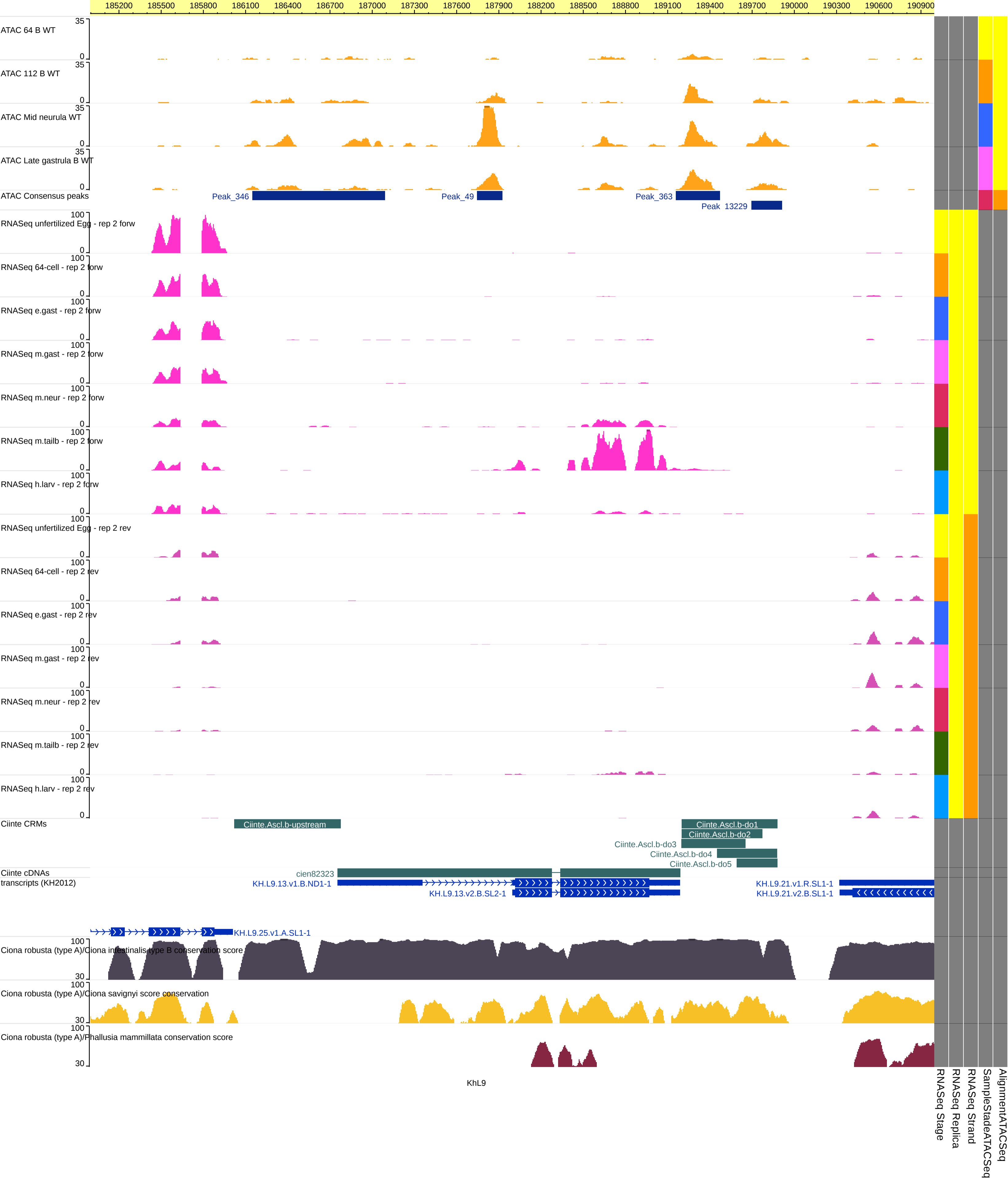

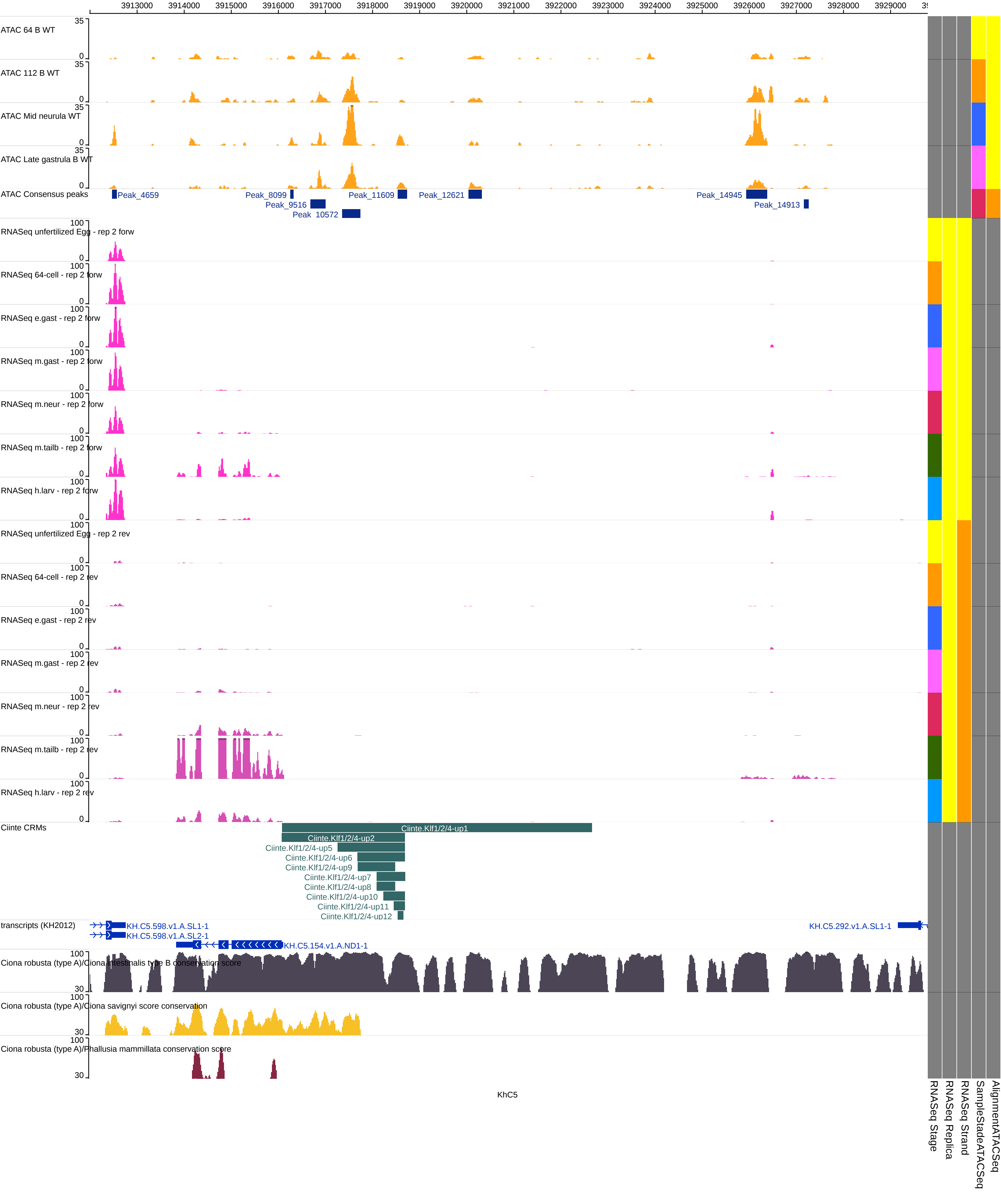

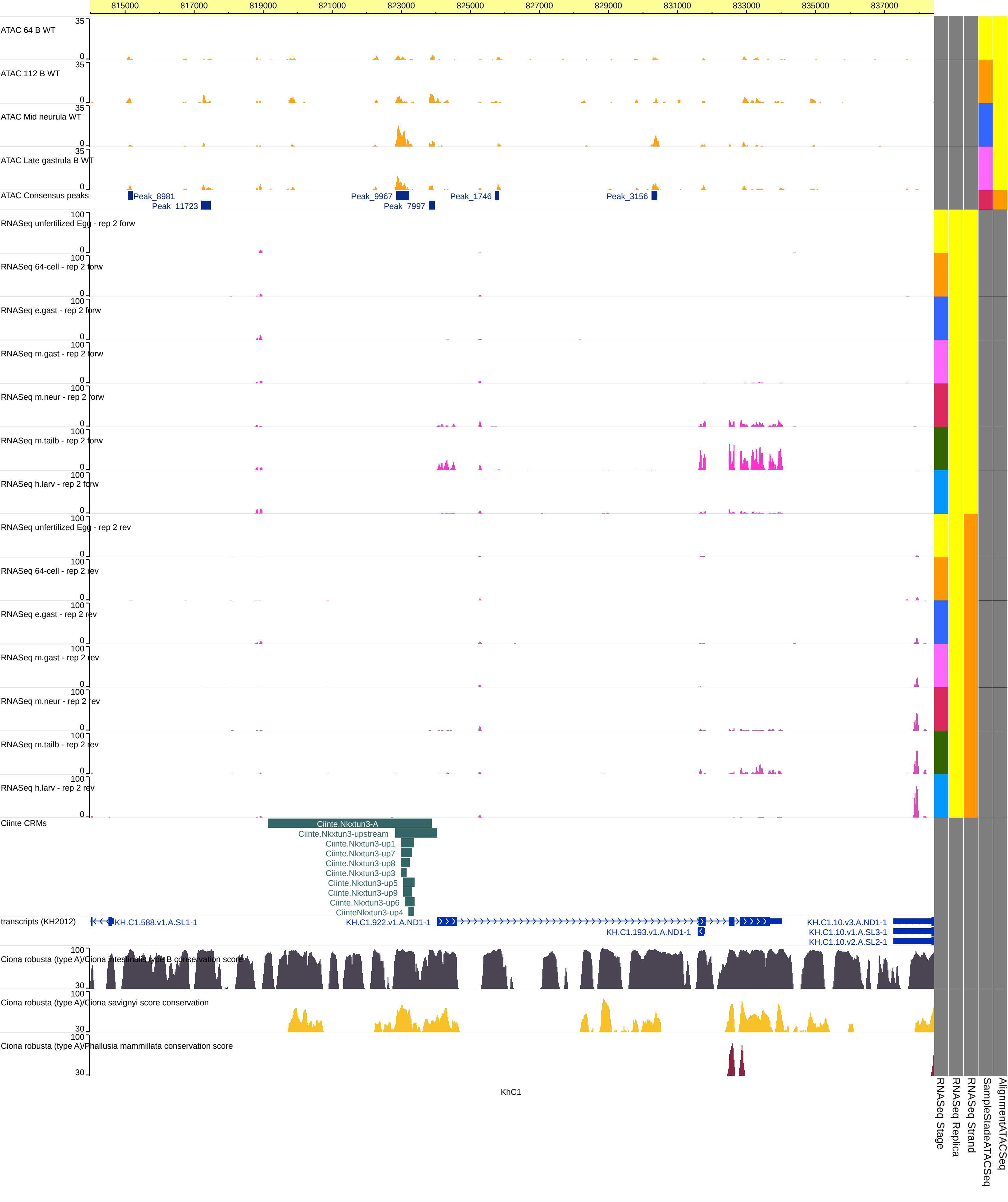

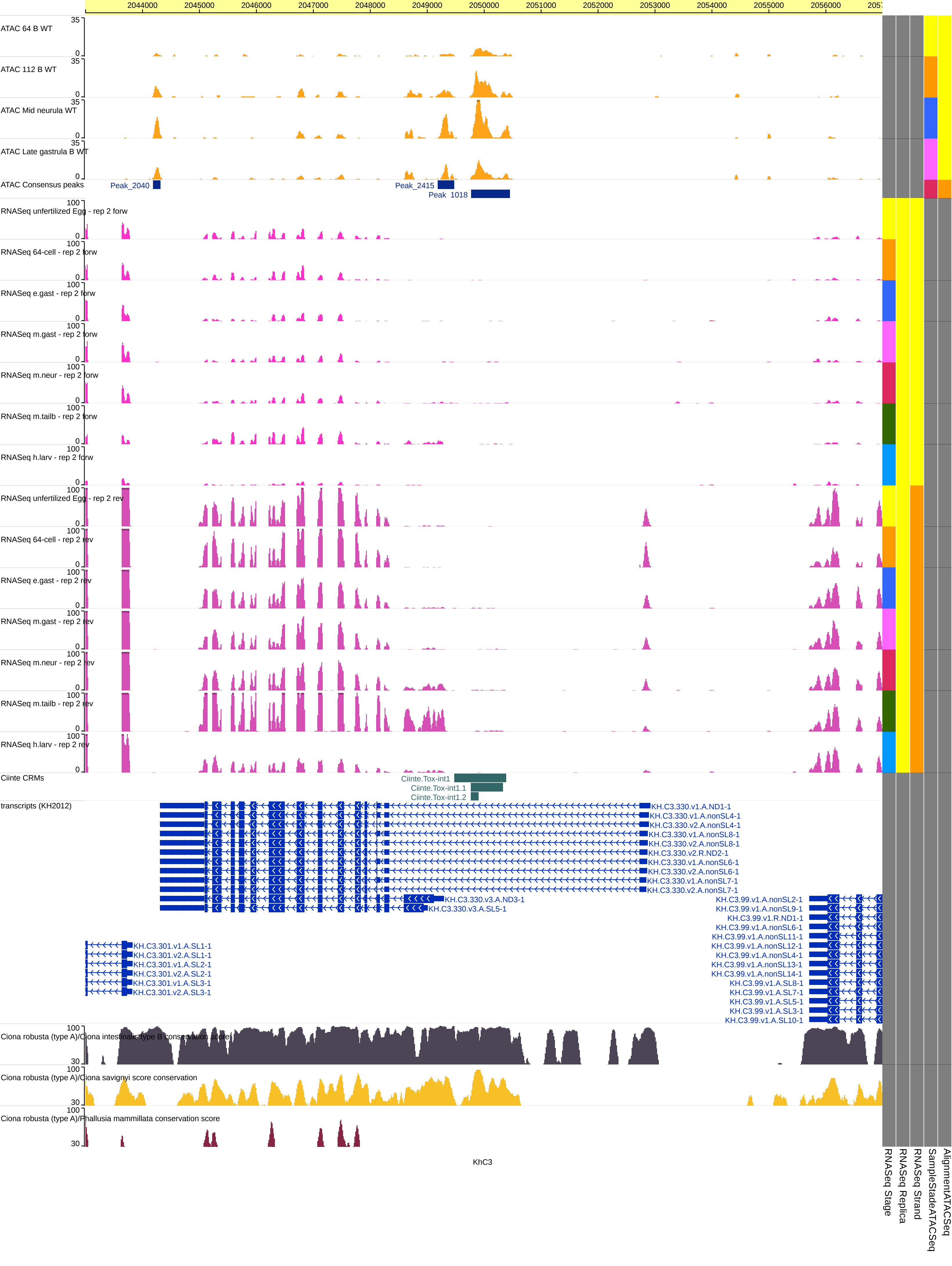

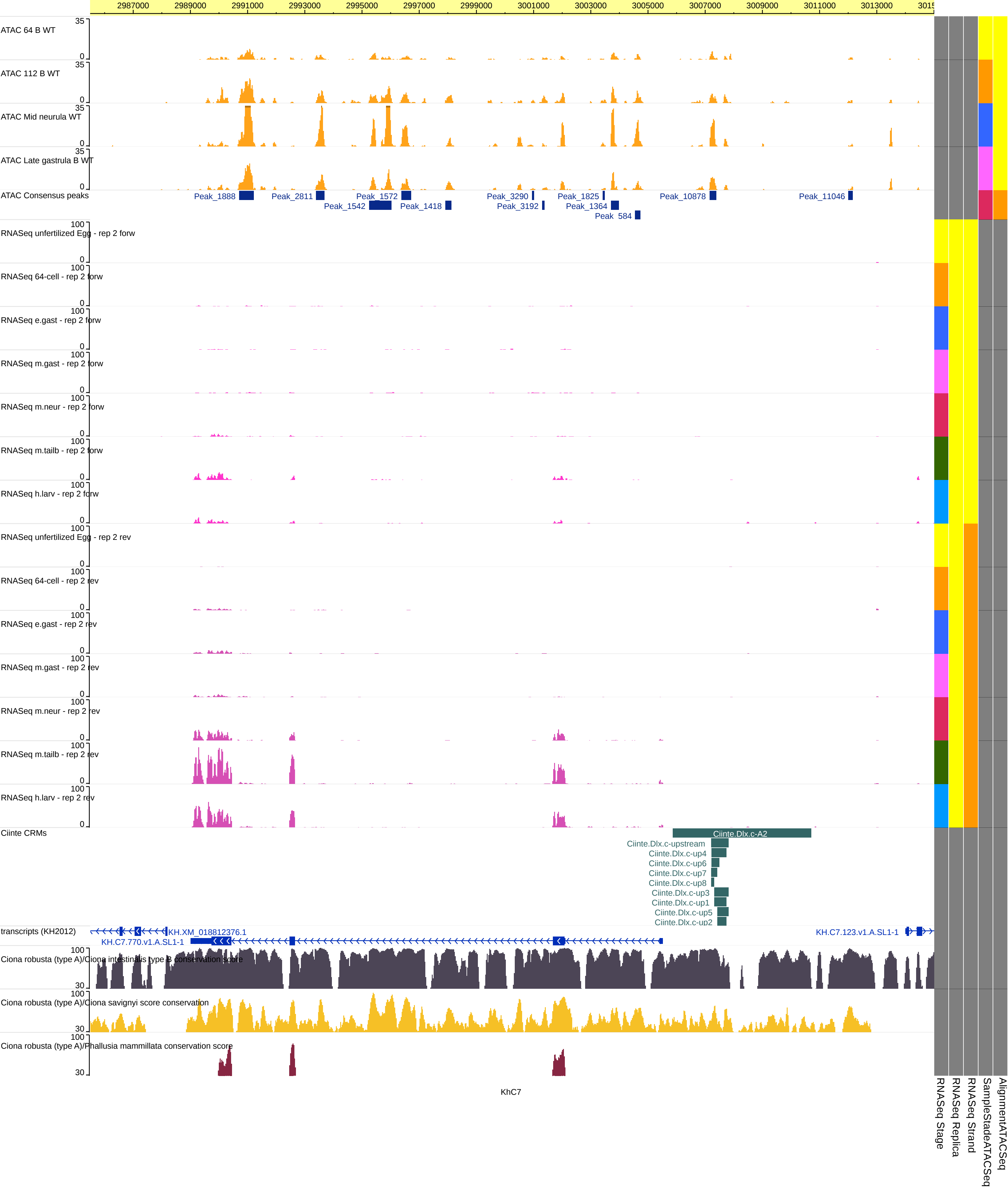

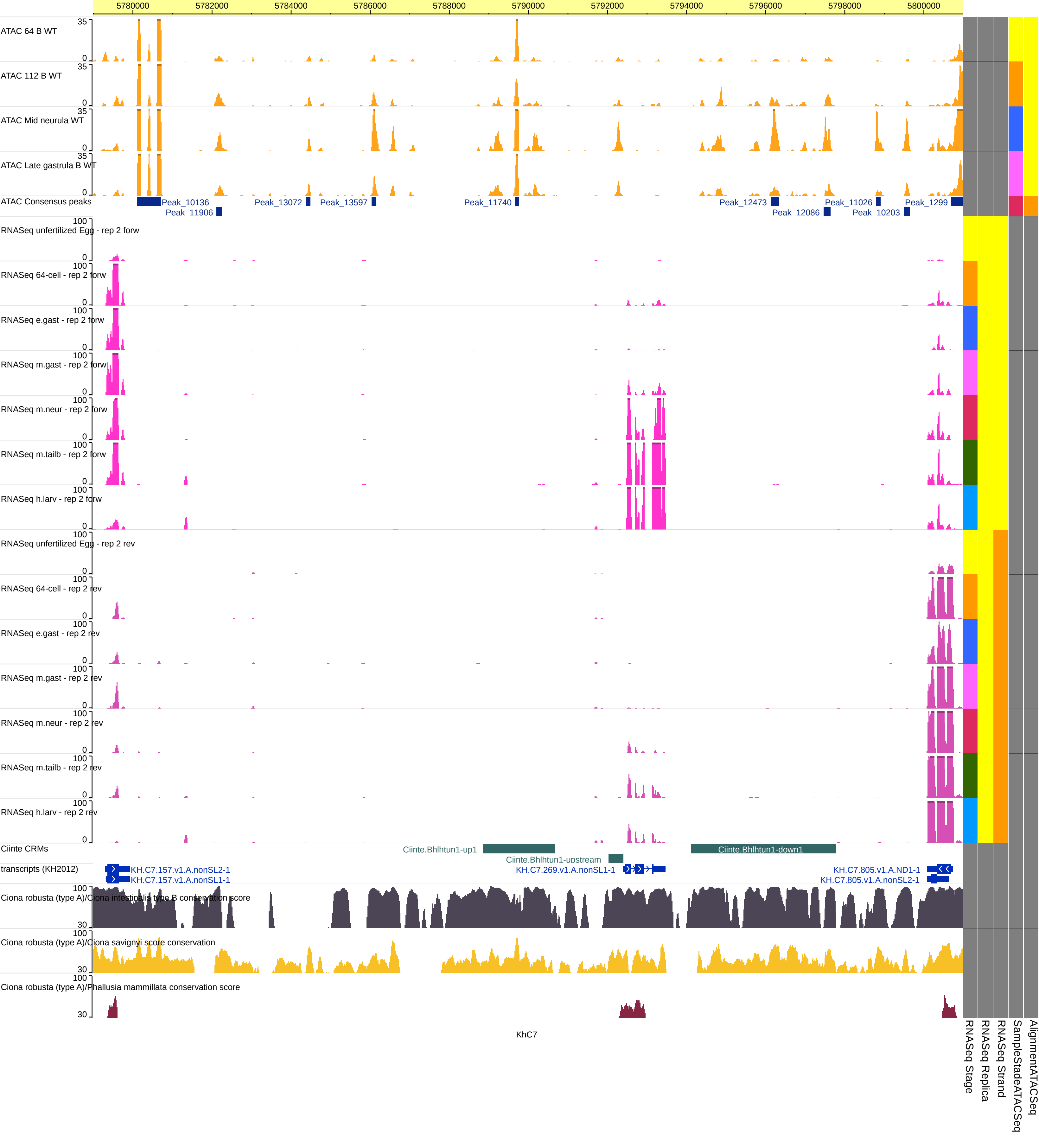

Supplement: Supplementary file 2. — Tested CRMs were added to the data extracted from the Aniseed website (https://www.aniseed.cnrs.fr/; Dardaillon et al., 2020). [file elife-59157-supp2.pdf]

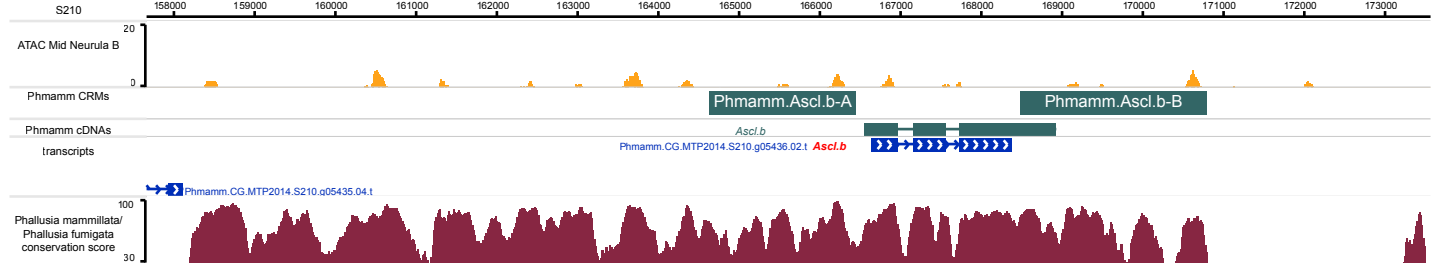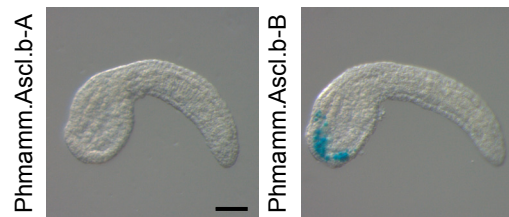

A (n=112, N=2)

B (n=165, N=2)

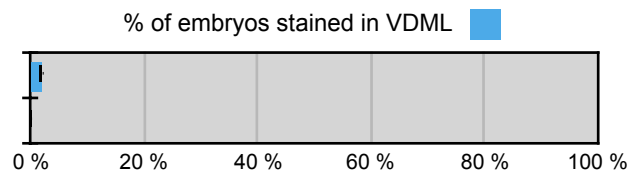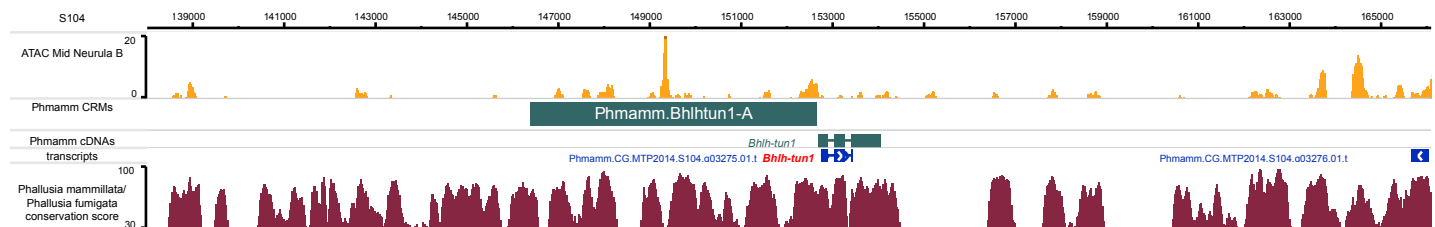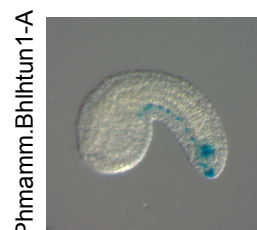

Phmmmm.Bhlhtun1-A (n=142, N=1)

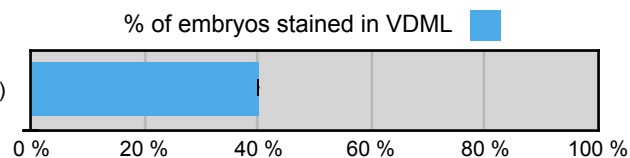

Supplement: Supplementary file 4. — Snapshots of the Phmamm.Ascl.b and Phmamm.Bhlhtun1 loci depicting ATAC-seq profile at mid-neurula stages, tested genomic regions, transcript models and conservation between P. mammillata and P. fumigata (from https://www.aniseed.cnrs.fr/ and Dardaillon et al., 2020; Madgwick et al., 2019). Representative examples of X-gal stained embryos at tailbud stages following electroporation of Phmamm.Ascl.b-A (no activity), Phmamm.Ascl.b-B (activity in palps and anterior nervous system), and Phmamm.Bhlhtun1-A (activity in notochord, endodermal strand and tail tip) into P. mammillata embryos. Embryos are shown in lateral view with dorsal to the top and anterior to the left. Scale bar: 50 μm. Schematic representation of the various constructs and their activity at tailbud stages in VDML (blue) (n indicates the total number of embryos examined, N indicates the number of independent experiments). Note that while VDML activity is rather robust for Phmamm.Bhlhtun1-A, it was not considered further since this activity was restricted to the very posterior cells of the midlines at the tail tip. [file elife-59157-supp4.pdf]

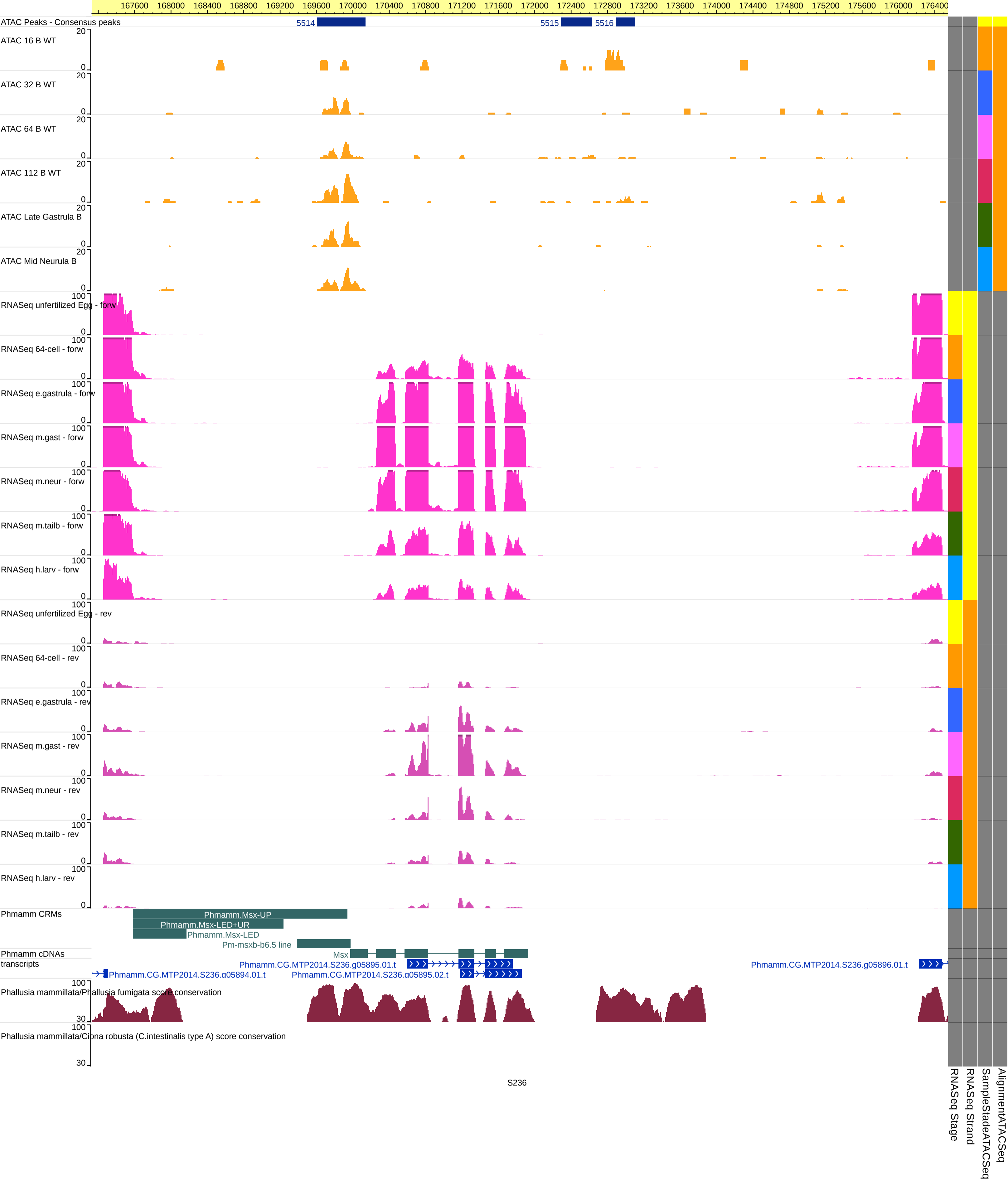

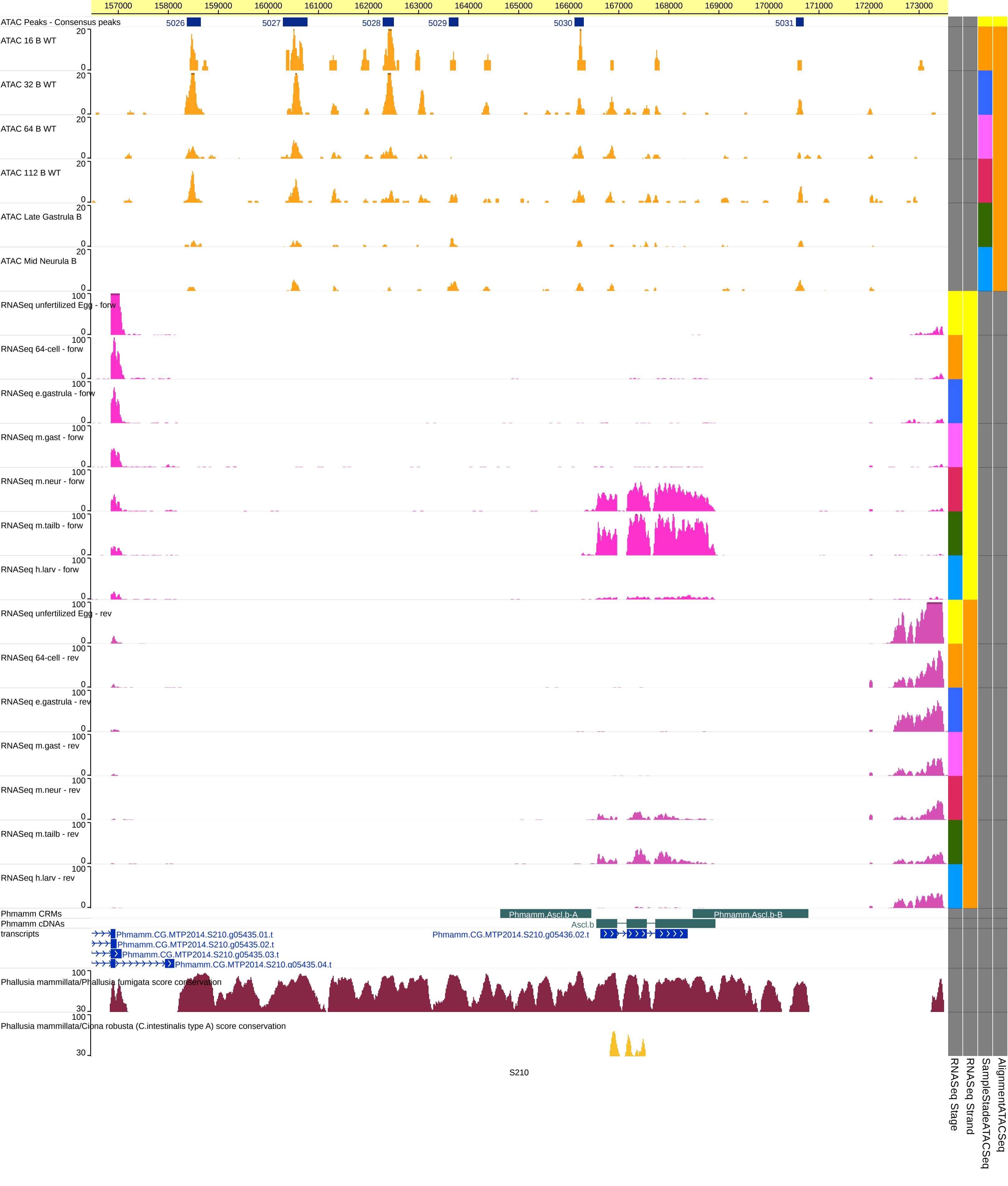

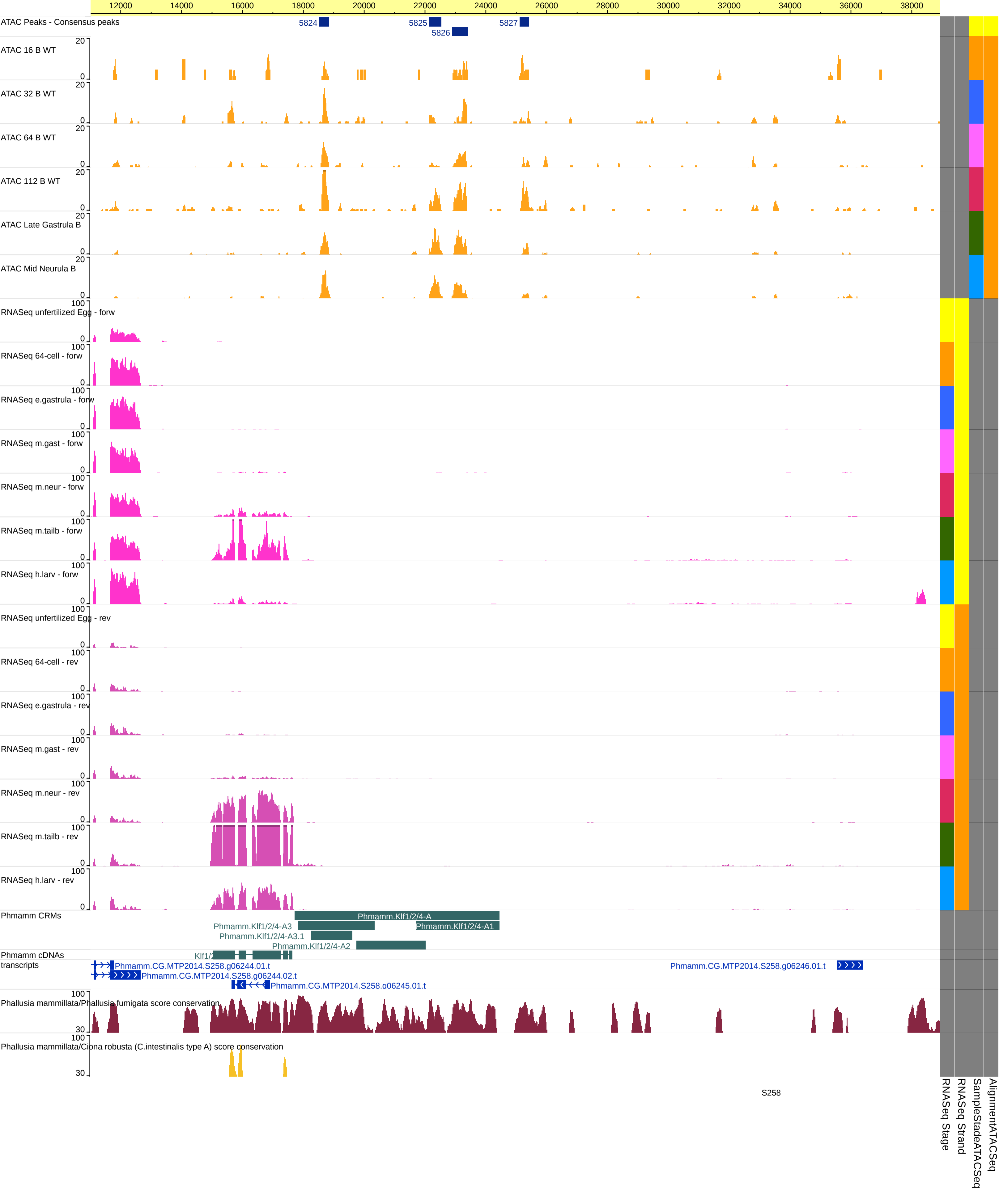

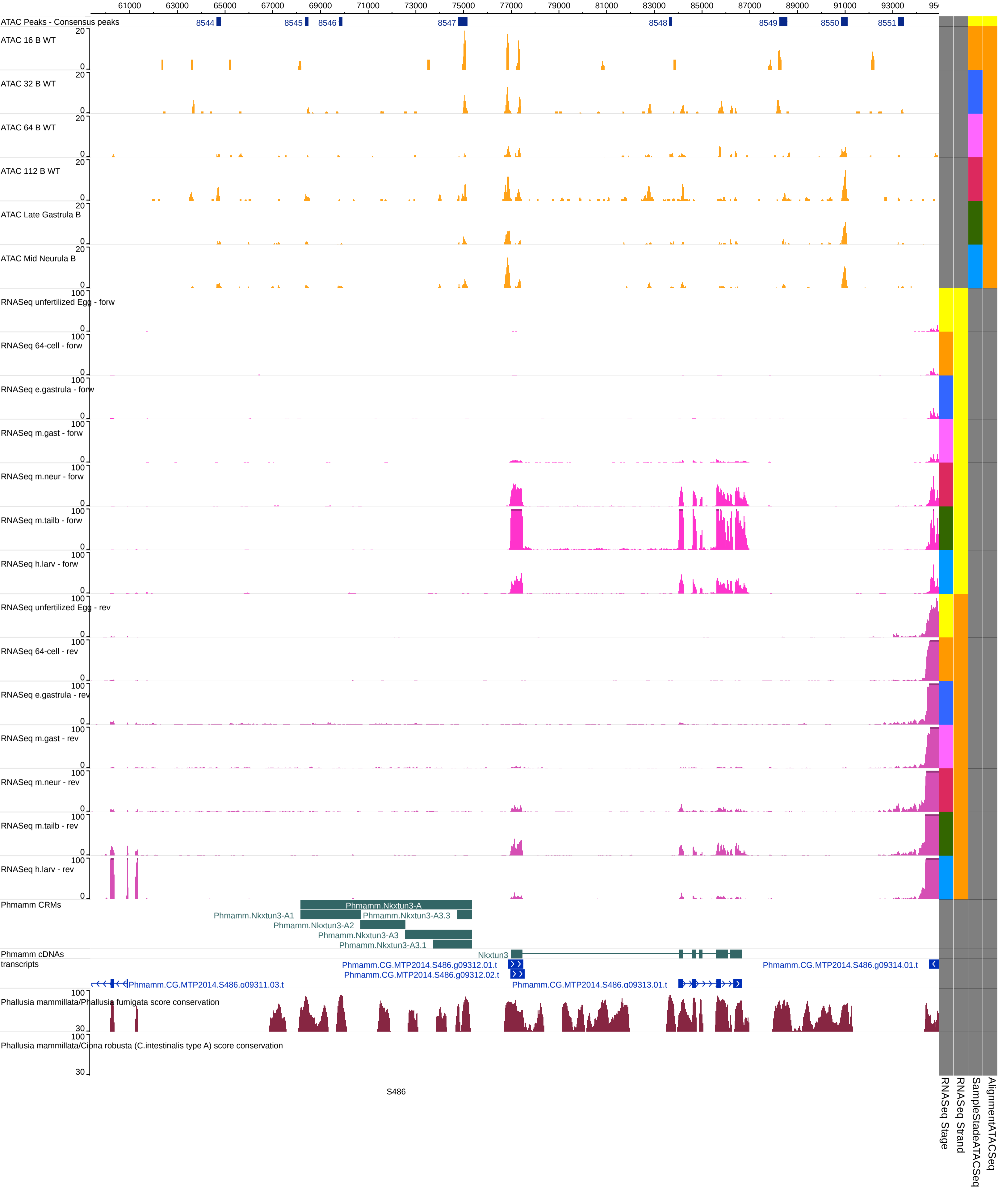

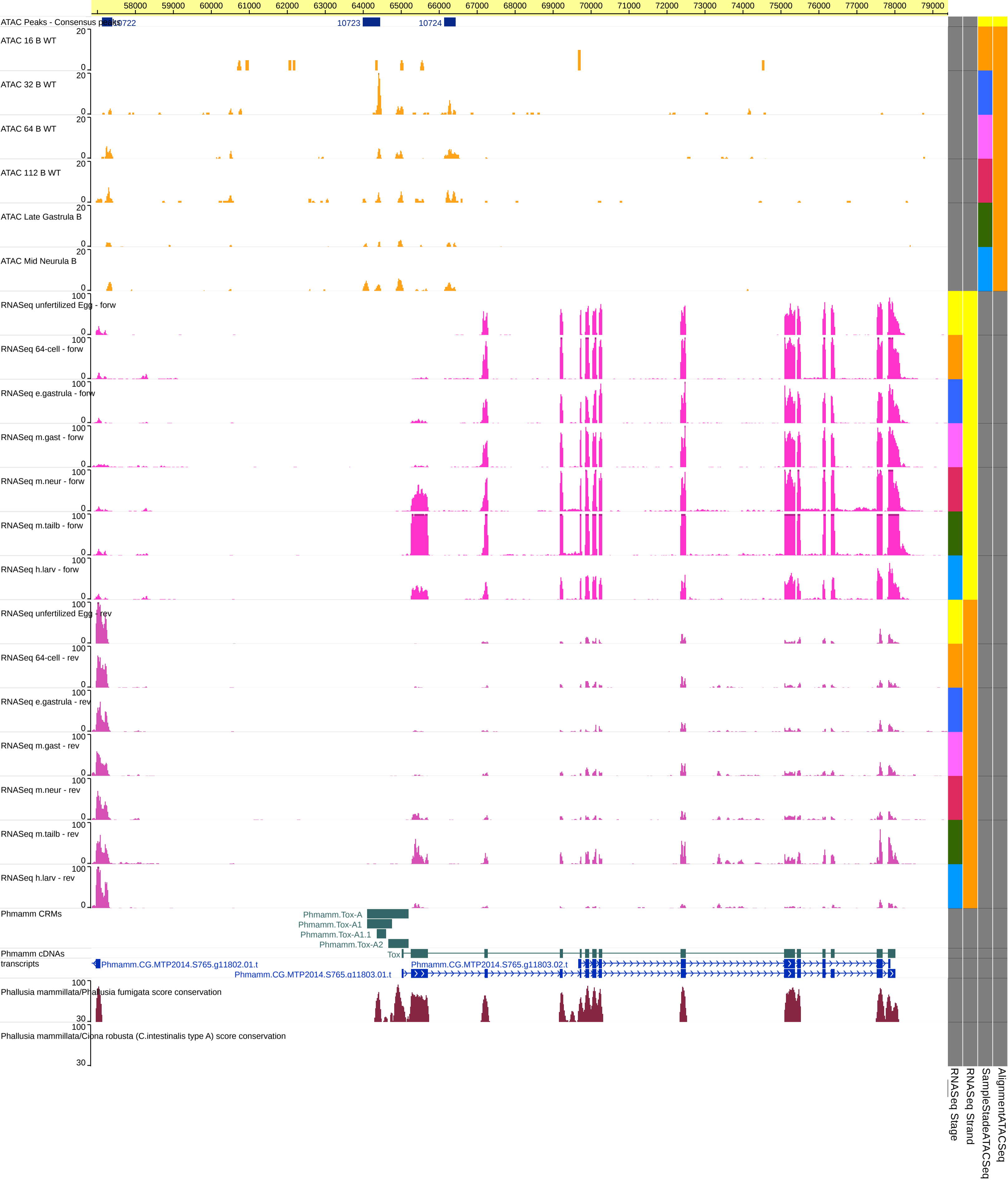

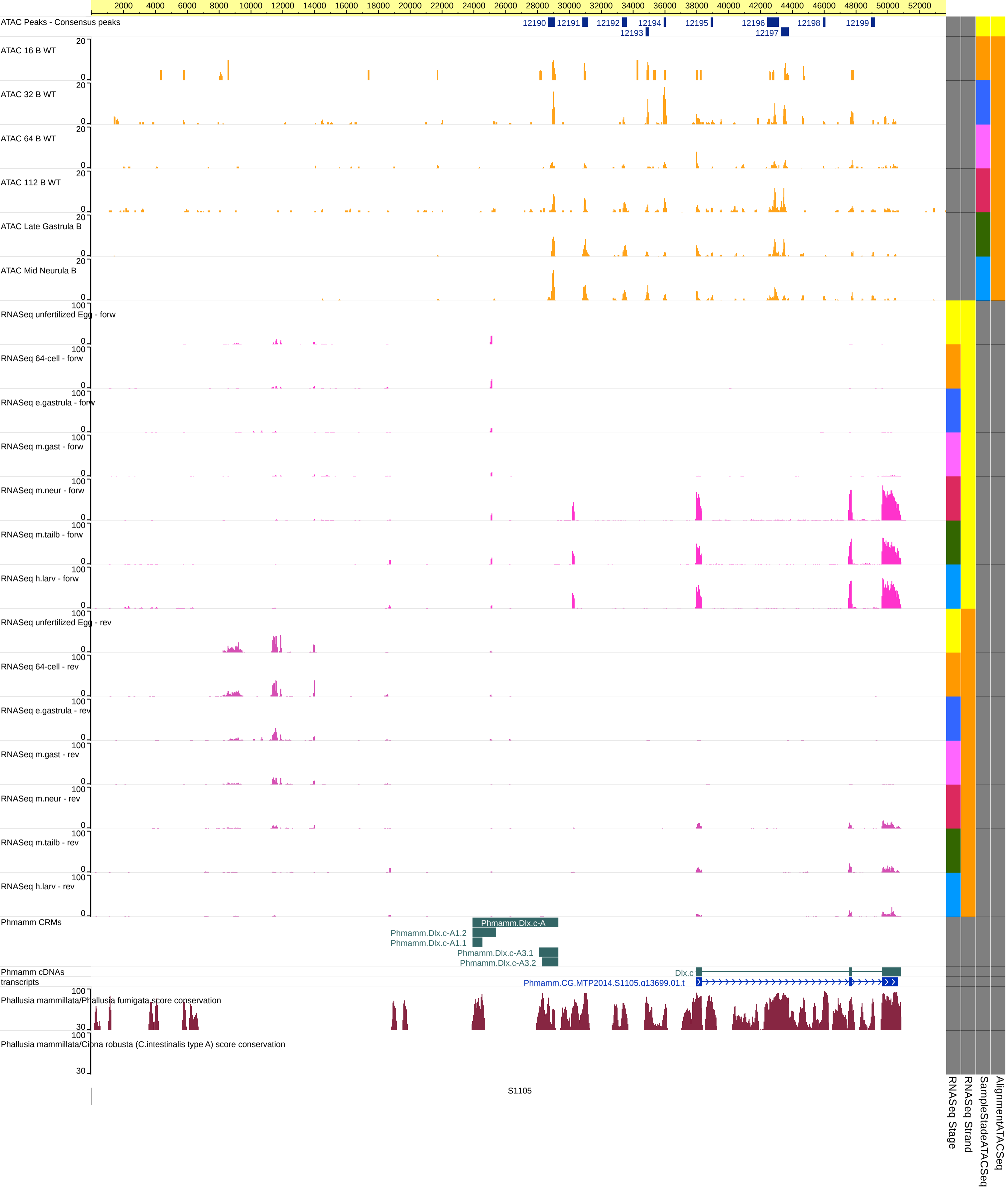

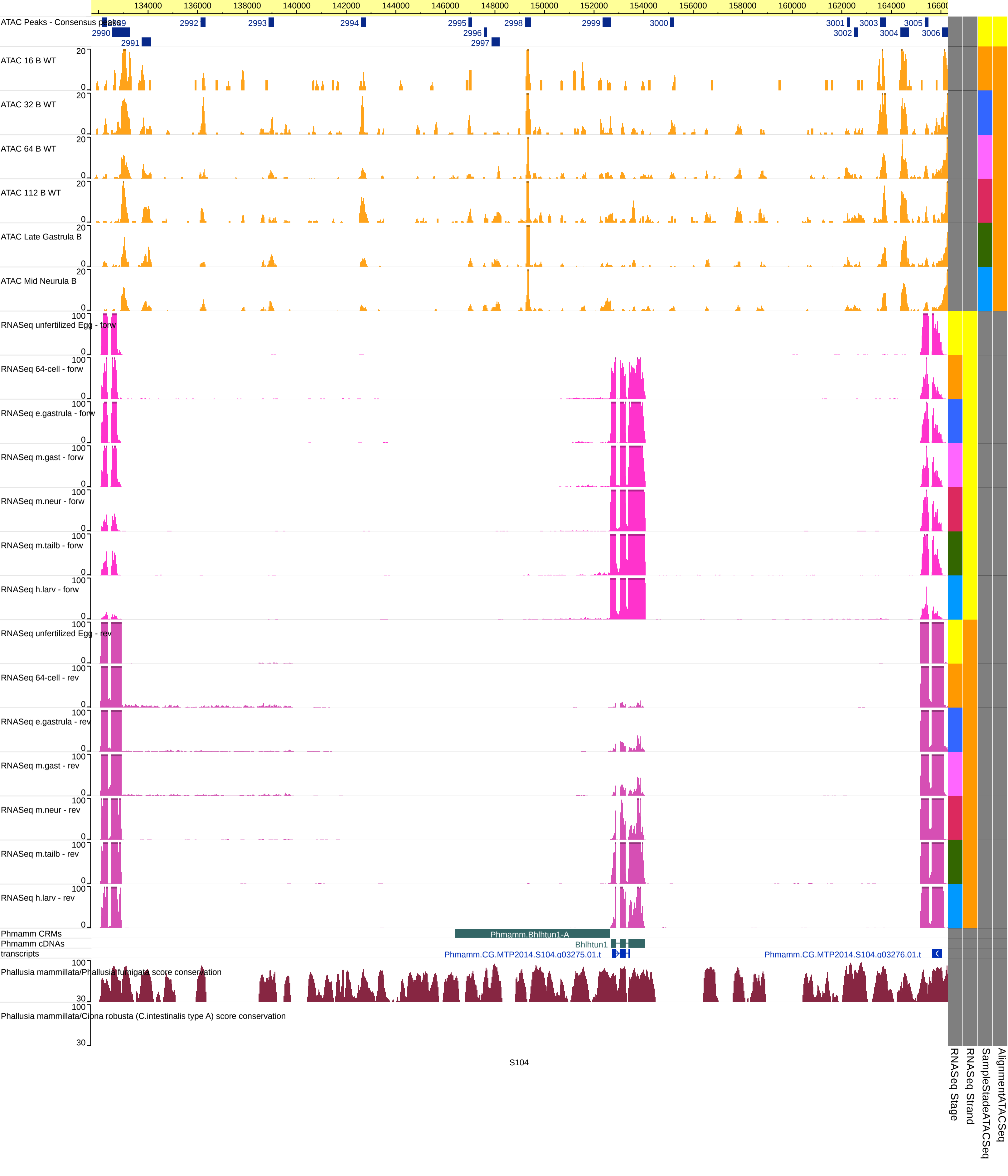

Supplement: Supplementary file 5. — Tested CRMs and predicted cDNAs were added to the data extracted from the Aniseed website (https://www.aniseed.cnrs.fr/; Dardaillon et al., 2020). [file elife-59157-supp5.pdf]

S143

27600 28000 28400 28800 29200 29600 30000 30400 30800 31200 31600 32000 32400 32800 33200 33600 34000 34400 34800 35200 35600 36000

Phfumi CRM

Phfumi.Msx-up

Phfumi cDNA

Msx

transcripts

[Phfumi.CG.MTP2014.S143.g01156.01.t](#)[Phfumi.CG.MTP2014.S143.g01159.01.t](#)

&gt;&gt;

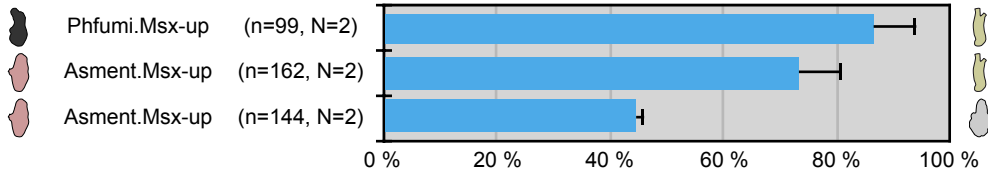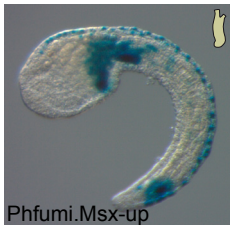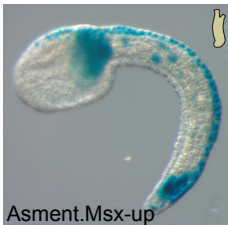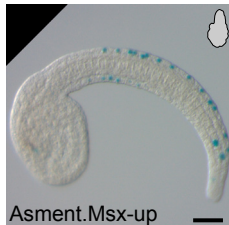

Supplement: Supplementary file 6. — (Top) Snapshot of the Phfumi.Msx locus. (Middle) Activity of Phfumi.Msx and Asment.Msx CRMs at tailbud stages in VDML (blue) of C. intestinalis and P. mammillata embryos (n indicates the total number of embryos examined, N indicates the number of independent experiments). (Bottom) Representative examples of X-gal staining at tailbud stages (embryos in lateral view with dorsal to the top and anterior to the left, scale bar: 50 μm). [file elife-59157-supp6.pdf]

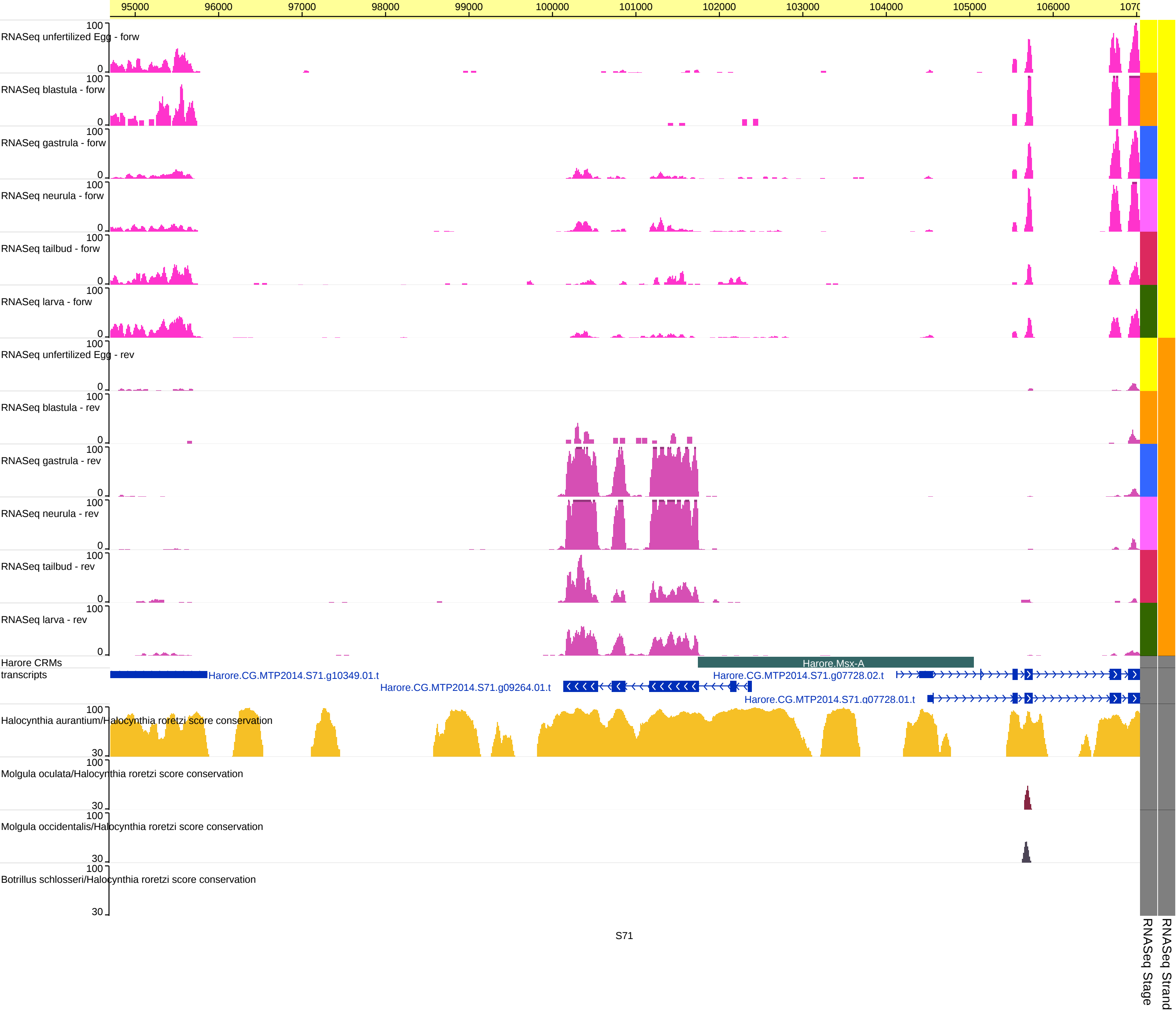

S71

RNA-seq Stage

RNA-seq Strand

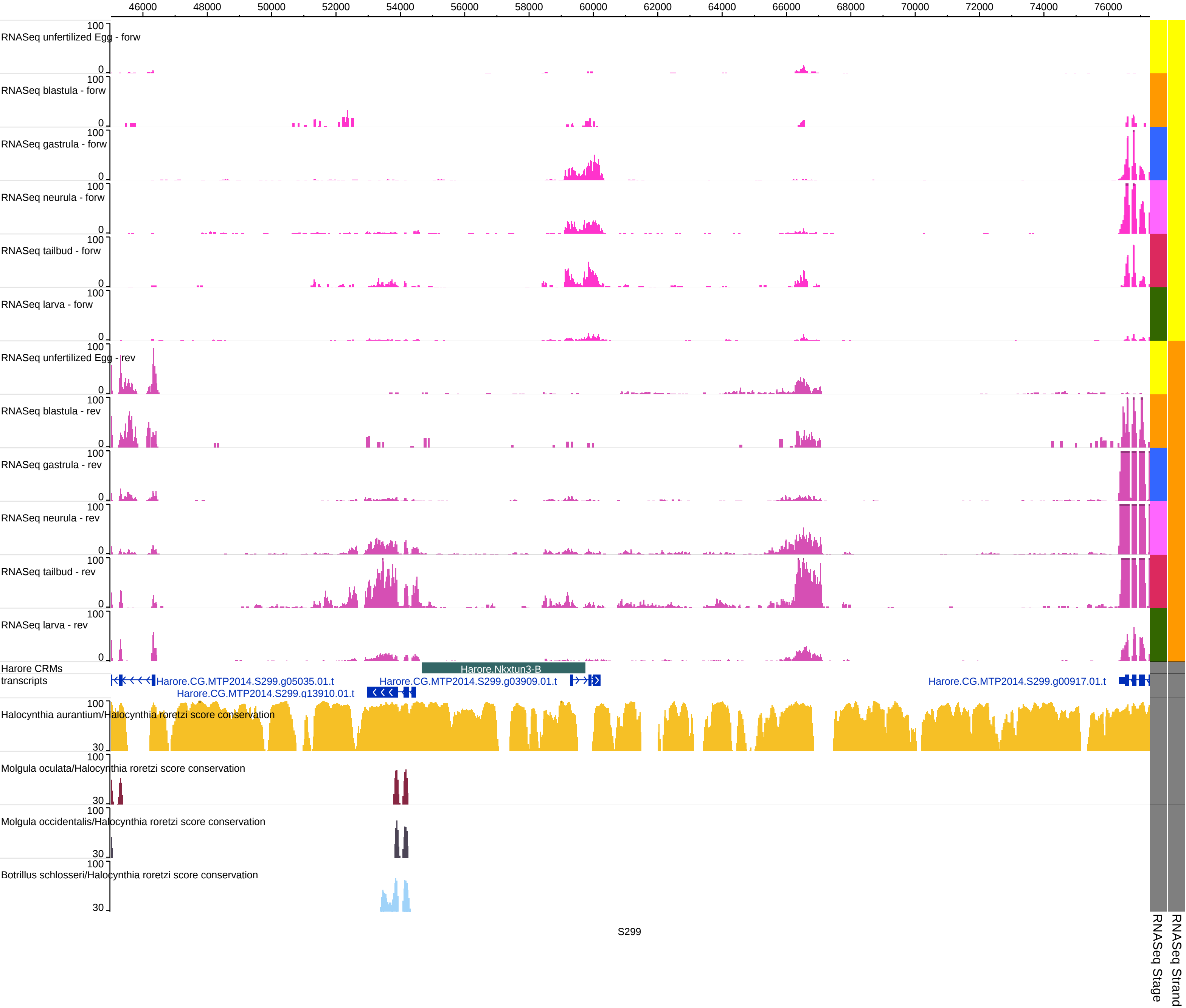

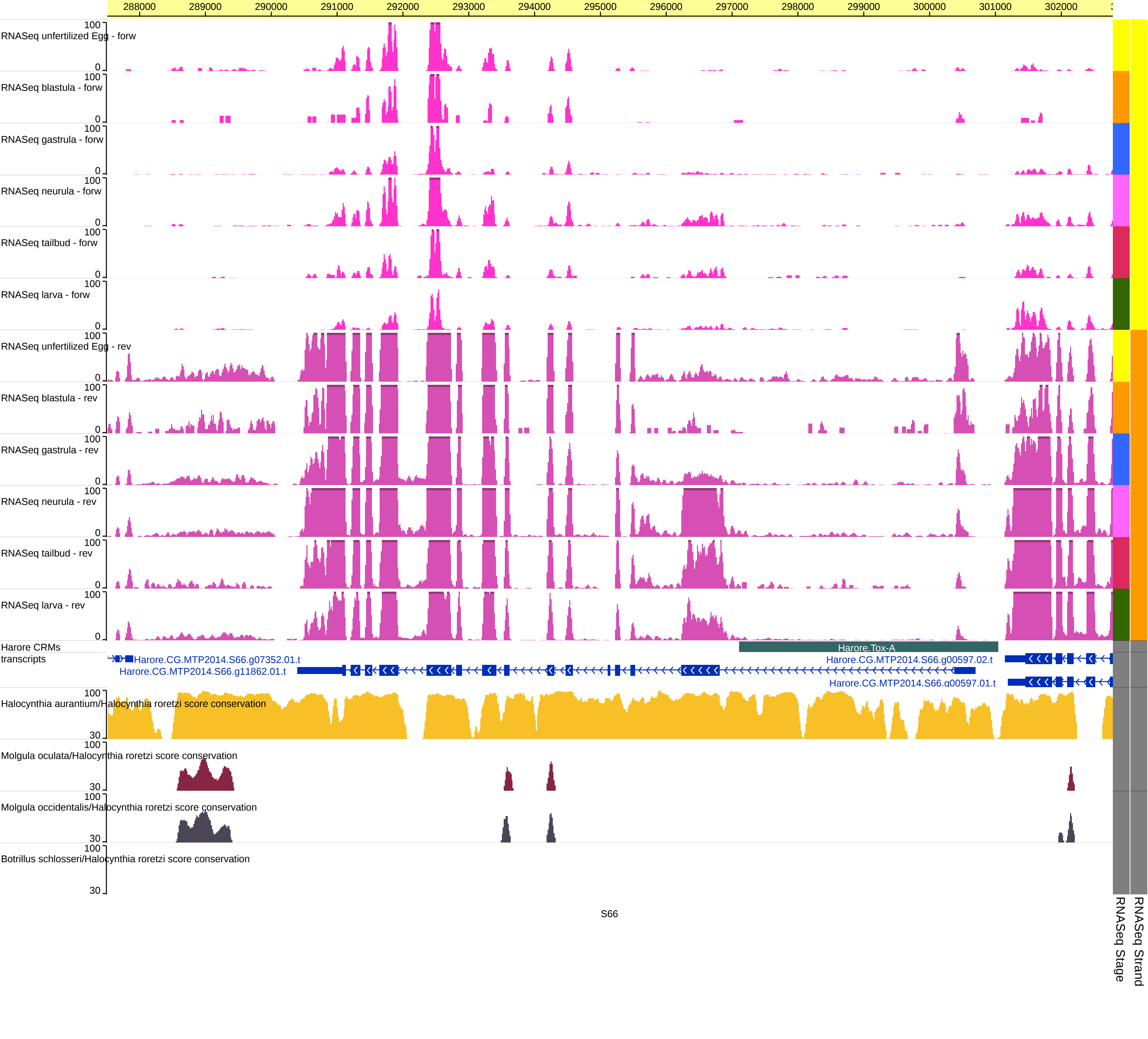

Supplement: Supplementary file 7. — Tested CRMs were added to the data extracted from the Aniseed website (https://www.aniseed.cnrs.fr/; Dardaillon et al., 2020). [file elife-59157-supp7.pdf]
