# Supplementary material for: Conservation of peripheral nervous system formation mechanisms in divergent ascidian embryos
Source: eLife. 2020 Nov 16;9:e59157. doi: 10.7554/eLife.59157 (PMC7710358; doi:10.7554/eLife.59157)
Supplement: Supplementary file 3. — (Top panel) Snapshot of the Ciinte.Bhlhtun1 locus depicting ATAC-seq profile at mid-neurula stages, tested genomic regions, transcript models and conservation between C. robusta and C. savignyi (from https://www.aniseed.cnrs.fr/ and Dardaillon et al., 2020; Madgwick et al., 2019). (Middle panel) Representative examples of X-gal-stained embryos at tailbud stages following electroporation of Ciinte.Bhlhtun1-upstream, Ciinte.Bhlhtun1-up1 and Ciinte.Bhlhtun1-down1. Embryos are shown in lateral view with dorsal to the top and anterior to the left. Scale bar: 100 μm. (Bottom panel) Schematic representation of the various constructs and their activity at tailbud stages in DML (blue) and VML (purple) (n indicates the total number of embryos examined, N indicates the number of independent experiments). Note that while VDML activity is rare, activity can be detected at other sites of endogenous Ciinte.Bhlhtun1 expression: anterior epidermis around the palps for Ciinte.Bhlhtun1-up1, and notochord and stomodeum for Ciinte.Bhlhtun1-down1. [file elife-59157-supp3.pdf]

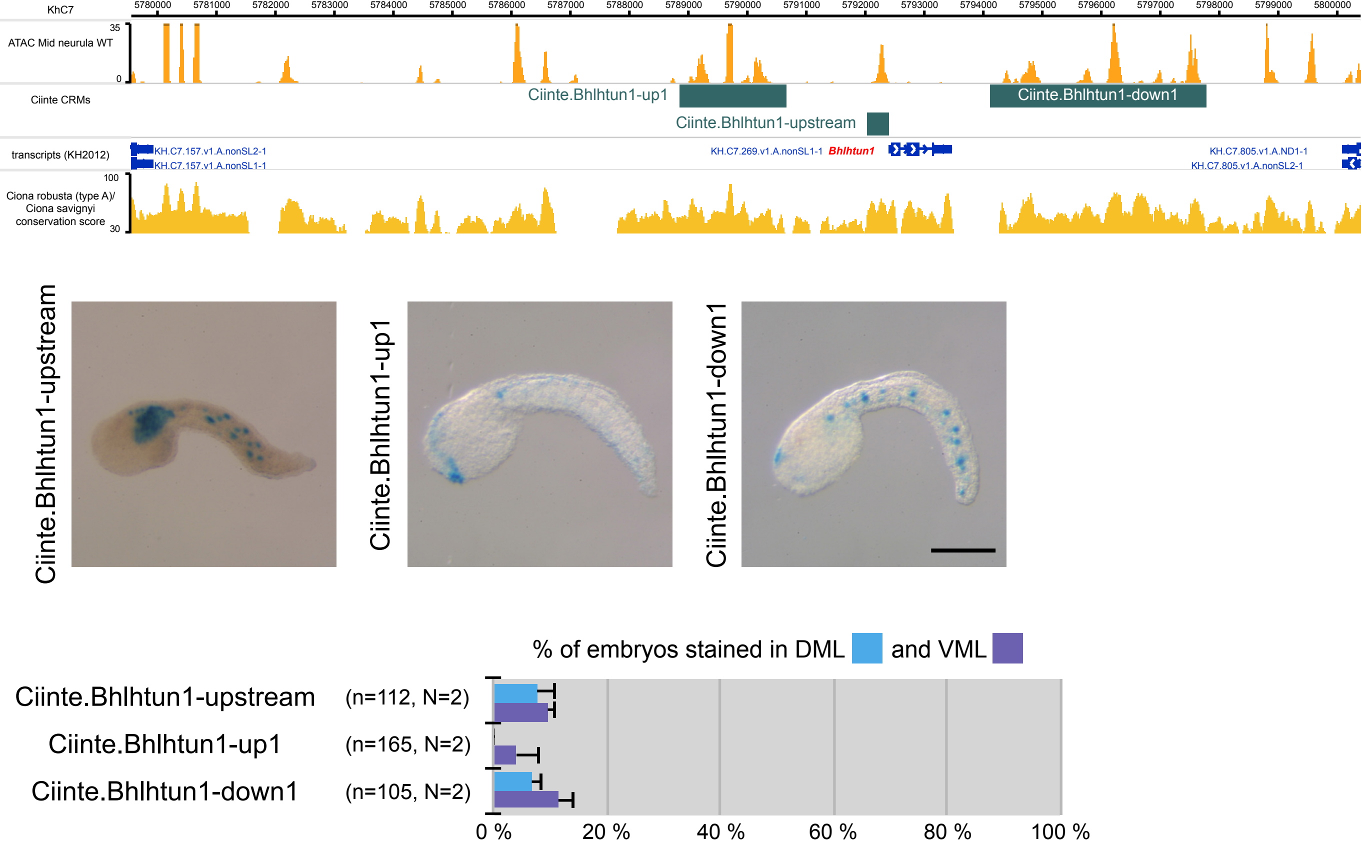
KhC7
5780000 5781000 5782000 5783000 5784000 5785000 5786000 5787000 5788000 5789000 5790000 5791000 5792000 5793000 5794000 5795000 5796000 5797000 5798000 5799000 5800000
35
ATAC Mid neurula WT
0
Ciinte CRMs
Ciinte.Bhlhtun1-up1
Ciinte.Bhlhtun1-down1
Ciinte.Bhlhtun1-upstream
transcripts (KH2012)
KH.C7.157.v1.A.nonSL2-1
KH.C7.157.v1.A.nonSL1-1
KH.C7.269.v1.A.nonSL1-1
Bhlhtun1
KH.C7.805.v1.A.ND1-1
KH.C7.805.v1.A.nonSL2-1
100
Ciona robusta (type A)/ Ciona savignyi conservation score
30
Ciinte.Bhlhtun1-upstream
Ciinte.Bhlhtun1-up1
Ciinte.Bhlhtun1-down1
% of embryos stained in DML and VML
Ciinte.Bhlhtun1-upstream (n=112, N=2)
Ciinte.Bhlhtun1-up1 (n=165, N=2)
Ciinte.Bhlhtun1-down1 (n=105, N=2)
0 % 20 % 40 % 60 % 80 % 100 %
