# Supplementary material for: Conservation of peripheral nervous system formation mechanisms in divergent ascidian embryos
Source: eLife. 2020 Nov 16;9:e59157. doi: 10.7554/eLife.59157 (PMC7710358; doi:10.7554/eLife.59157)
Supplement: Supplementary file 9. — Predicted cDNA sequences for genes in C. intestinalis, P. mammillata and M. appendiculata: transcripts models from RNA-seq data and ESTs sequences were used to build the cDNA sequences. Open reading frame is highlighted in bold. Sequence of the genomic region Asment.Msx-up isolated from A. mentula. [file elife-59157-supp9.docx]

>*Ciinte.Ascl.b*_cDNA (cien82323 clone)

ACTGGCAATATGATTTTATTTTGACTGATAGTGACCTGTTCGTTGCAACAAATTGATGAGCAATGCTTTTTTATAATGCCAACTTTGTACAAAAAAGTTGGAACAGGTTCGTGTCGAATTGAAATTCAGGATTCTTAGGTATTGTTACGTCACATATGTCGAGAACAAGCGTATCATTCGATCACGTGGTAATCGAACCCTCTGTTACGTCACAAGTGATATTCAACGTGGGCGGTCAAATGTTCCAGAATCGAAATAGTTTCAACTTTTAATCCGCTTGGAGGCCAGATTAGTGACGTTTGTTTTAGAACGCTGTAAACGCGATTGACGCGACGATGATTCTTTATTTCGGATTGATGGAACAACCGCTAGAGTCGCTGCCGGGACGAATCGCTTTTACGCGAATTAATATTCGACAAAATATCGATTTGTTTAAGCGCGAACAATCGCTATTGTGACGAAGCACGAAAATCGCTGCGACCGCGGCAAATATTTTCCTCTGTGACGTCACATAGACATCTATAAGCGAATTTTCGTCGAAAATAATTTTTGTTGAAATTGAATTAAAACCCGATGTCATGCGTGTGGATTGCACCTAAGGGCACCTGGCAAGCGTATGCGGTCACGTGGGTGACACGTCACGTGATTATGACGTTTTATATCGCGTTGAAATGATGTTTCGATGTTGTATATTCGACTTTCAACTGTGAATATGATATCATAATGGGGGTTGTGTTGTGGAtAAGTGGCGTTAGATCGGATGCCCCCAGGGTCGATACATTCAACATTAAACTTGGCATAGAGATCGTATAATCCGGTTTGACAACAAACAACATAAACATCTTATAAACTCCGGAACTTAAATTGTTTTCGCGTTTAAATGAATCCCGTTTGTTACGTCACAAACATACGGAACGATACAATTAAAAACTTAATTAAAATTATTTACGTCACAAATAATTATTTTGTCATGTTCGAAAACGGTATGTGAGTGTTGTGACGTCATAAACGCAACCGGAAGTGCCCCACGTCATCAAGAATCGATGTTTTTGCAAATTGGGAATCTTCCATCGAAGTTTTGAGGTTGTGTGGTGAGATGAGGAGATTAAATAAAAGAGAATAAGAAGAGCAAAGAGTGAGAAGGGCGCGTGGCCGAGGTTTCTCATCTTGCTCCTGACCAATCAGCGTCGAGGAGTGGTTTGGTATAAATTGACGGCACGCGACACTCTTGTGTCAGCCCCACTTCTCACGTCATAATGGAAGTTATTCTCGCGTAGTGACGTGGGGGTTTGTGATGTAATATTTGAGTTGATGACGTCATAATAACTAAGAATTCTCAGGTTTTGTTAGTTTTGAATAAAA**ATGGCGACCGGAAGTGACGAACCGCGGTCGAACGCGATAATATYGATGCCGTTCAACAATAGATGGAAGGAAGGTTTGGGGGAAAATAATCCAAACTTCCGGGTCGAGATTCGGGGTGGGGTTGGGGGGAAAAACCCCACCAGTGTAGCGAGGGCAAATGCTTGGGAACGAAGGAGGATTAAAAACGTGAATTCAGCATTTGACGAGTTGAGACAACATGTACCTAACGGTGAAAGAAATCGAAAGAAGATTAGTAAAGTTGATACTTTACAATCTGCTATCGAGTATATCAAAGCATTGGAAGAACTTGTGCGAAACCGGAAGtCCAAAAGtGACGTCATTAATAAAGAGAACGCTACAACGTCAAACGCTGTGACGTCACAAGACGATGACGTCATGTTCGTAAAGGAAACTGAAGTGACGTCACAGAGGAAAAAAGATTCGAAATCTCCGGTAGCGTTAACGGAGTCGATGTTGAAGGCGTTTGACGTCATGTTGCAAAAATGTACGTCACAATCGAAGACAAAAGAAGATGATGACGTCATAAGGATGGATTCAACGAGCGACAGCGGTTTCTCCGAGATCCTCTGTGACGTCACAAGCGGCGGGGAATCGATGACGAATTTACCGCCAAATATTCCAGAATCCCCGATCTTACAATCGAACAATTGTGACGTCAGTTTTGAATCGCTGCAGGGGTTTCCCCCAAGTTACAACCCCCATAGGTTCACCCCTTACCCCACATACGTGGGTGAGTGGCCCCACATGCCCCCTACCCCGGGGGAATTCCCGCCAGTGGACCAAATTACCCCAACCCTTCAACTTCCGGTTGGATTTTCAAACGATTTtCCCGCCAACGACGCTGATTGGTtAAACCACAATCATTTCTGA**TCACCACGTGACCTTAACCGACCAATCATAGCCTTTCACTGCTTTGCTTCTTTATGACATCATCAATCCGCTTTGTGACTTAATAATTTTTATTCGAAATAAACTTCCATTACCGCGCGCGTCTATTTGTAACAATGGATGAGAAAACAAACAAAGCGACTATTTGTCCCATAATTACGTCATAATGGTGAAATATCAATAAACAATCGAACCGCGAAAAAAAAAAAAAAAAAAAAAACCCAACTTTCTTGTACAAAGTTGGCATTATAAGAAAGCATTGCTTATCAATTTGTTGCAACGAACAGGTCACTATCAGTCAAAATAAAATCATTATTTGCCATCCAGCTGATCCC

>*Phmamm.Nkxtun3*_cDNA

AAGAATGGATAACAACA**ATGAAGCAAGGGACAATACTTGGGAAAACGAACATTGGAAGGAAAAGCTCTTTTCCCCAGCGAGAGAAATGTCTAAAGGAAAACGAAAGCCGAGATAAGACAAGGAATGATAGGAATTTTCATCCTTACGCAAGGGAACGGAGACAGAGCAAAGAGTCCTTCCGTCATTTGATGCCTTATTTCCCGTCAAGTATGATATCCGGCCCGTTCCACCCCGAGACTGAGCCCGCCCCGGGAAGATTTAACCACCACAACTCTGCTGAAAAGGCGGAGTCGGAAAGAGGATGTATGCCGAGTGATAGTGATCGCGCCTTGCCAAGGGTTGGACCGAGCCAGACAGAGAGACTGGCAAGCGTTGGCGCCCAACAGCCGGAAAATAAAATGGTGGACGCGCAGCAATTGAAGGGAAAGACCGACATCGAACAAAACGCAAGGAAACATCAAGAAAACGTGCAAAATCAAAATGAACCCACGTCGATGAAAACTTCAATTTCGGACGAATCTGCTCCGATTTGGCCACTGGGCGGAGACGAAGAGCACGCGGAAAGCAACGTTGACGTCACAGAAGATATACCCAAGCCTCGTACGCACTCGAGCGTGTCGTCAGTGTCCAGTGACATCACAAAGGTGTCAGAGAAAGACACAATGAACGCGTACTCCCCGCCCGGGGAAGTAAAGAAACGTCCGCGCACCGCATTTACTCCCGACCAAATAAAACGACTCGAATCTGAGTTTCATCGCAACAAATATCTGTCAGTGGGAAAAAGAATGGAATTGTCCAAAGCCCTAAAGCTTACAGAGACCCAGATCAAAATCTGGTTCCAAAATCGTCGCACCAAATGGAAGCGGGAATATCTGAGCGAATGGGAGGTTTGGATGCATCAGAACTACTACGCAATGCACGGGCTGTACGGGGCTACTGCAGCAGCAAATGCGCTCGCAGCACACTCCAATCCGATGAGCGCACCGTTTGTACCGGCACCGGGAGGTAGTTTACCGAGCGAGAGGCCTTCTGCGTTCGCAGCCCCAAGCCAAGTCGCCGGAAACCCGTTTTCGAAACCGGGCTTCTACCCTGGCGTGGGTTCGATGCTTATGAGAAGCGGGCAGTTCGGAAATGCTGAGGTCAGCACAAAAGATGAGTTTATGAAACAACCGCCCCAAGGACAGATCGGGATTCCGCGACTGCTGGCTCCGATTCCGAATTTTACGTCGCCGTTTCAAGGCGGCGCCCTTCCCGGTGTTCCGTACTACATGTCGACGCCGGGCGGGCGGCCCCATCTGACGCCGCGCGACGCCAACACTTCACCGCCGATGCGTCAGACAAGTGACAGTCCGTCGTCAAGCGAACGAAGCCTAAACACCTCCCCGACTGTAGGTGGTTCCCCACGCACCGCGTCATCTAGCCCTGCAGCTCAATCTGCCCACAGGTCGAGTGTGGAAGAGAAAATAACCTGGAACAACGGGCTGGGATGCGAAAAATTCCCCGAAGGCCTATTCAACCCTATACAGTCCGGTCAGATCCTTCGGCATGGAGCTTTTAACACTACAGGAGCGCTGACTGCGTCTTCAAGCGGCTTTCTTCCAAACTTAATGGCCAATGGGGCCTTGCTGCGAGCTTACGGGCTTCAAGCTAATCTTTCGTCCGTTCGTCCGCTCCCCCATCTGAATGGAGCGCCCACTATCGGGCAATTGGTTGTCCCCCCAACTGTGCCCCTTTCGCCACAGTCATTACGCAGATAA**

>*Phmamm.Dlx.c*_cDNA

CGGAAAGCCAA**ATGAGTGCTTATGGCTACAGCTACGAACCAACCCCGGCATCGGGATATCTCGGCAACGCTCGCGGGCCTTCGGGCTTCGGAAACCCGGTCGGAGGCAGCTACGGGATGCCGGGCTCGACTTATGCGTCGCCTTACGGTGGGACAAGTCAGCACGGAGCGATCCAGGGCATCACAGCGTCTGTAGCTTCCGGGGTCAGCCCAGTTTCGAACCATCATCCTCACAGTATGCAGACCTCTACTTCGGGTTACTCTGCCCCTCCACCCCCGCCTCTTGGGCCTGGGGCCGGTGGTTTCCCGGGGTTCTCGTCGGCGTACGGACCCCCAAGTCACTCGTCGTACGGGGGCCTGATGAACCAATACCCGAATTGCGGGAGCCTAAGCGACGCCGTTCTACACCAAGATTCAAATCACGTGATGGGAAGTGACGTAATGATGACAAAAGGAAAGAAAAAGAAAATGCGAAAACCTCGAACGATATACTCGAGTCTCCAGTTGCAAGCTTTGAACAGAAGATTCCAGCAAACTCAGTATTTGGCTTTGCCAGAACGGGCCGAACTCGCCGCCACGCTCGGACTCACGCAGACCCAGGTGAAAATCTGGTTCCAAAACCGACGCTCCAAGTGCAAGAAGCTGATGAAGCAGGGAATCCACGATAAAGACAACCCCATGGTGTCGCCAGGAAGCAACAGCAGCAACACAAACAACAACGCCGGTTCACCATCCTCTATTACGTCACCTATGATGTCATCCGTGCCGCCTACTACGTCATCGGATCTCGAAACGCCGGGCAGTGTGGGTCCAGCGCCGGTTGGGGCCGATACAGGGGTACCTGGGTCGATGGGAGGCGGAACCTGGACTCCAAACTCAACAGACGAGCAACCCTCACCACCCCCTCCTGACGTAATGACCCCCAACCGCGGTATGACGTCACCACCGATTACGTCACCGTATCATATGATGACGCCGCTTCACAGTATGACCACATCGATGAAAAGCGAAATGTCGTCGCCCCTGCCCTCGAACGGGGGGAGTCCCGGTATGTTACCACCCTACACCCACCCTGGAGGAGTGTCACGTGATATACAAGGCGGCTACATGCCCCAACCCATGCACGGGGGTCAGGGCGCCATGATGCAGGGTAATTTCTGGTACTCGGGTTCAGAACACGACCCTATCGAACCCCGCCCCGCGATGAGAGAAACAGCCTACCTTAAATAA**GGCAAAAATCAAAAAAAATCATGATTCTTTTGCACAATCCCCTTCTCCTTACCCCGTTCTGCATAAGAGAGCATGTAAATACGTCATCTCCGCTATTTATCCCCCTACCGGAAGTGACGGGACTCGCTTCCACAACGGTCTGGTATCGTTCCCTTTTGTTTAGTCATAATGCGCCATTATTACGTCGCCATTCAATCTACGGTGTGTAGTTGATTTCTATGTTGATTCGTGCCTGATCCGCACGAGGTCGCTCTTGAACATGTATTTGTTTGCTCAAGAGATCTTTTTATATTATGACGTAGTAATAGGCCGCTATGACGTGACTGATAGCATTATCAGGCGAAAAGTTTGTTTTTGTTGCAAAGCAATTCATGGTTAGAGTTATTTCGCGCAAATTTATCGGAATTCGACATTATAACGAAAGCGCGTGATTTCTATGGCTTTGGCGGTTACCATTTAAATTTTGGGCATTTAATTTACTTTTTGGTGTTTCGAAGTAGTTGGAATTTTCCTTTTCGTGTTTTTTTTCTCGTTTCTGAAGCAAATAAATCCTTCATCGCAAT

>*Phmamm.Ascl.b*_cDNA

AATTTTTTTTTG**ATGGTGGTTGCGGCTGTAAAGTGCAAAAGTAAAATTTTAAACCCACGACAAATGACGAAGGATAGCAATATGGCGTCACTCCACGCATCTGCCAGTGAACAGAACATGTTTCAAGTTGCCTACGATAAAACATGGAAGGCTCAAGAGTGCACAAACAAACAGCAAAGCAACGGTGCGCTGACCACCTACACTCTGCACATTAAGAACGATTCGATCACAGCCACCCCGGAACCACTGACAGAGGTCCGCCGAGGGGGCGGTGTCGCAGGGAAAAATCCTTCCAGCGTTGCCAGACGGAATGCCCGCGAACGCCGGCGAATTCGAAACGTGAACAGCGCCTTCGATGAACTTCGGCAACATGTGCCCAACGGCGAACGGAACCGCAAAAAGATTAGCAAGGTAGACACGTTGCAGTCCGCGATTGAATACATCAAAGCGCTGGAAGAACTTGTCCAAAACCGACGCTCAAAAACAACAGCCGCAGCTGAAGCCGAAGCCAGCAAGGAAAACGCGCCAAAGCTGAACAAATGCACCAAGCGAAACGAACAATCGCCATCGCCCCCTGACACACCTGAACACCGGAATAATGGCTCCGATTGCGATGACGTTATTTTTGTAAAAGAACTCAGCAATTTTGCCGAATCCCCAGCGCCAAACAATGAGCAAAAATCGGCAATGGAAAGCAGTGGTAAAAAGGTTCAACGCCCAGCAGCACTCACCGAACCCATGCTTAAAGCTTTCGACGTTATGCTTCAAAAGTGTGCTTCCGAGAGAAAGGCGTCTGCTTCGCGTCCCGACGGTTTTTCCGAGCTTTTCGAAGACCAGGGCGATACCAGCAGCGAAAGCTCTTTTCGGCCTCGCCAGTCTTCCTTAGAACTAGATCAAAGCCCACCTAGCATGGATTTTGTTGCGTCACCAATTCACAGTGAAACTTTACAAGCAACCGTTGCTGAAACATCAGTTGGAGGGTTCTTGCAGCAACTTGAAGATTTCACGCCGTCGTTTGAACAAATCAACACACCAAACAGCCAAGTATCAACATTTGAGGACGCTCAGTTACCCGACGAATGGCAAAAGCCTTTAGGCCGATTTCAGCAGTTTTATCCTGAAGCCATTGCTGAAAATTGCCCAGCACCACAGATAAACTACTTACCGTTGCCCGCAAGCAATGCTAACTGGCCAAACGGCGTACAGGAAATGGAACATCAAAATTTTCATGAACCCTTTGATGTTCAAACCGTATCCGTGCCGAGTGGCCTTGACCAAATCAATCCAACTCTGGACTTTTTTCCCGGTCCTGACAGCAACAACAACTATTCCTTTGTTGAGACTGCACCAACATTTATGAACATGAACGCCAACCAAGGCTGCGAACAACCGACATTCAGCCAATCGCTTTTCTCCGCTCGAGGAGTGGATGAGAGTTTTGCTGTCGCGCAGTGGTAA**TGCCTAAACCACGCTTTTTTACGCTTTTACCATTTTCCTTTGTTATACCACACAATCATAAAAGCTTGATAATCCAACAATCATTTGTAGTTTAGTTTACCAAGTGCCTATCTGCCATCCATGCGCAACAACTTCCAAATATACAAGTTGACACACTGCCTTAACCATGCTGCCGTACTGCTGTCCTAAAGTTTCTAATATTATCACTGCCAACCTTGTGCGTACTAACTTTTTTCTTTGCTCTAGTGCCTAAATCGTTAAGTCGTCTCATGAGACAAACGTCACTGCCAACAAAACGTACATGTTTGAGATCGTTTTAATTTATCGCGTGCGTACTGTCCATTTATGTCAGCAATGAAATCAAATAAGAATTTATTTTGTCTATTGCAAGGAAATTTACAACCAGAAGCCTGCATGGACGTGTTGTTTTAGGTTTATGCTATTGAAACATATAGCTATAATTTCCATTTACCGCCGTTCGTTTTCTTTGATTCTAAGTTTTGTGTCCGGGCGTTTACTTGCTTTGCGCTAAATAAATTCACAAATAAATC

>*Phmamm.Klf1/2/4*_cDNA

TACACATCAACAGCACTAACTCAGGTTGCTTAATTTGAAGTGA**ATGTTGAGCACTGATCCGATTTTCACTCACGGATCCTGCAAAATGATAAAGGTCGACACGAGCAGCAGCGAAGAATTCGTGCAGCCGTTCCCCCAAATCGGGGGCCTGCTTTCCCGCATCGATGACCCAGTTCACTCGGGCTCAGTGGGAACGGATGTCCTGGCTGACCTTGAGAATTTATGGGACGAGAGCCCGGACTGCTTCGATACAATCCTCAACACCTCAACTAGTGATTGTGACAGCGACGGAGCTGTGTTGTCCCCTGAGCATTTCGGTTCAAAACTCGCCCCAATCGGCGTGACTTTCGGTCGGGCATCCAAACAGGATCGCCAGCACGGCGTCTTTGTTGAAGGAGATGAGGCGACCGATAGTGGAAACGGTTCTGAGTTTGGTGGTAACGGTAACCTTATTGCTGATTTTCTCAGTCAAGGCTCAGAAAACAAGGAAACAGTGTTACCGAATTTCTACACTGGTTCGCACGCGCAGCAGCCTTCAGACGCAGTGAAACCATCATCACTACTTACGCCGACTCTTTCGCTAACCGGTGACAGTTTTCAGCCTTACAACTTCGCCAACACTCAACCCAGTCCCGTTGAAGAAAATAGAAGCCTGAAAACTGTGTTCAAAGTCGAGCAGATATACGAGCAAAGCAACATGAAGTTACATTGGAACGCTCCATCTTTTCCACAACCAACAAATCAAGTTCAAGTATCGCATGGGCTGGTCACGCCACCAGTGAGTCCTGAGGAAAACGACCAATTCCGAAAAAGTCCCTTCCATGTGTTACAAAACCAAGTCAATCGACAACCTTCACCAATAGGTCACGAAAACAGCAATCCAGAGCAGTTCAGGCCCTTTAATCCGCCGCAATACACCTCTGCGACTCATCAGCTCCACCAGACGGCAGAAATGTACAACGCACAGCCCAGTTTCGACTTTCAAATGAAAACCGAGCTGCCCAGGGGGCTTGATCAACAGGGAGGCCAGTTTCCACTCAACCTATCAACAATGCATTACCAAAACGCAAAACATCACAATCCGGACCCACAGGTCTGGAACAATCCAAACCATCACCATCACAATCAAAATCAGGTCGTGTTTGATCCCCGATGTGCCAACGTCTTTGTTCACCACAAGGTAAAAACTCAACCTGCCGGAGACAATGTTCAGCTACCGGGCATGGCGCCAAACTTCCCAGCAAAACCTCCTACGACCGGGAAACAGAATGTTCCTGCTGGAGGGGAGAAAACAAAACGAGGCAGGAGATACTGGACGCGAAGGAAGGCCACCCTTCATACTTGCGACTACATGGGATGCGGAAAAACTTATACCAAGAGCTCGCACCTTAAAGCCCACATGCGAACACATACAGGCGAGAAACCTTATCACTGCACGTGGCAAGGATGCGGCTGGCGGTTTGCGAGGTCAGACGAACTCACTCGACACTACAGAAAACATACAGGCCATCGCCCGTTCAAATGCAGTATGTGTGAACGCGCCTTCAGTCGATCAGACCATCTCGCTCTGCACATGAAACGACATATGTGA**GATCCTGAAATCTAAAAACGATGATCGAGAACAACCGCGCGGTGACGCTGTCGAGCAAAACGAGGAAGAGTCACGTGGACGAGAGACAAGTCATAACGTTGAAGGACGAAATTTGTTTACCTATTTCGCTTTAAAAGTGCCAAAAAAACAGCTTATACAATCAAACGCCTTGACAGGTGCTTTACAGAGCGGTGCTACATGTTGCTAAATGTGGACCAGAATCAAGTGTGTTTTTATTCGCAGTTTTTGCAGGAACAAAGATTACAGCAGCCGATTTGGTGTTTTTCATTTGTCAAAAAGTTCGTTTTTTACTGGTGCCAAGAACCCTGTGTGCTGTATTGAGTGATGTGTGTGCGCACTTTGTGTTTTCTATTTGTTTGTGGCAAAGTTTCACAAAGCACAGGATATGTTTGTGTTCATGATGTTGTGTTTATGTGCGCGAGAGATCGTTAGTGAGTTTTTTTATATTGAGATATTTTATTCTTTTTTACCGCTTTGCACTGCCGCTTTTAACGCCTCGTGTCGTTGTCACCTTTTTATATCGTCACCCCTGTTTTGTG

>*Phmamm.Bhlhtun1*_cDNA

CGAGCTGAACAGTTTACCAATACGACTAGCATTTCAAGCAAAAGCAGAACGTCAAA**ATGGTAAAAGCTAGCCCATCAAAGGACTTTAACAAGCACTTTAGATGCAGAGACATTAATACTGTTACCGAATCGAATATCAAAAGGAAGACGGATTCAAATGCACCGAGCCATGTCCGAAAGCTTAAGGAAAAGAATGAATTACGAACCTTGTACCGTCAATTGAAAGACGTCATCCCTTCATGTAAATCGAAGCCAGTCACGAGTCTTGACATTGTACTTCGTGCTGTGGATTACATCAATGAGTTACATGGCATGCTCGACGAACAGCAGCCCGAAGTCAATGCAAACGATAGCAGCGAGCAGCGTTTCGCCCGCCAGTGCATGCAAATTGCAGCCAACAACGCGAGAAGCATGTCGTTTCACGATATTACAAACGTGACAATGTGCACTGAAATGCGCACCACGCCTCGCATTGGTTGA**TCACCGTCACTGAGCAGTTACATCATCAATGACTTGTCAATGTCATCACAAATTTACGTCATAGCTGTGACGTTGGAACGAGAACGAAGAGGGATTTGCGAACGAGGCTCGTTCTCCACGAGTGAGCAAAACCCGTTGCCAGGGAGTGGCATGACGTCATACGGTGACGTAGACAATGACGTGCATTCGCATGTGCCTTATAGACAAAGCCTTGTGCCGTATCACCGGCCAAATTTGCCTTTATGTAGATTTTTGTTGTGTAAATTTTGTATAGTTGTATTTCACCAAGGGCCAAACCTGATTGCGATCAATTAACCACCGTCCAAGCCTTAGATTGTATGTTGTTTTGTGTAAATGTAAGGCCAAATTTATCTCAGCGACCAAGATTAACCCTTCAAACACTTTTTCGAATCTTCCTGTGCCGTTTTTAGCCTTTCCGAACATTGTCCTGTCGTGTGTCTTCCATTAGCGCCTTGTTTTATTGTATTGTACATACACAACGCGTGTGCTGTAATCTTGTTCGCGTTTTCAAACGACATTCGCCAACAGTGCCTTCTCTTGTACACTCTCGCGTATACGCTCGGCGTTATGCAAATAAACCTATACATTCCCG

>*Phmamm.Msx*_cDNA

GTGTGACGAAATTGCGGCATTGTTATAATCGAAAATCGTTTTATTACAATTTAATGAAGACTGTCAGCGGCTGAAGGAAGCGCGGATAATTGAAGATAGTGAGAGAAGAACGCCAAGAAAAAGGGCTTTCTGTAGGCGGAATTTTTTTGTACAAAAGCGCG**ATGATCTTGATACTCCAGTCACATTACGGACCAACTAATTTGTTTAAAATGACACTACTTTGCGAAAATTCTGCTTCGGAAAATTCCGCAACGGGACTTATACCGTTGCCAACAGCAGCATCAGAAAGTTCTTTAAACGAAAACGTATCGTCCAATGATGATTGCAGTTCCGAAGAAGAACCTTCGCCGACGAAAACGTCCATCTCTAAAAAAAATCGTGACAAAAGCAATTTCAGCATTGAATTTCTTTTGTCAAAACCCACGCGATCAAGCGCTGTCGAAACAAGGAATCATATCCCATTTAAAACAAGAGTGCCATTGATTGATTCCTACACACAATATTATCCGTGGATGATGACAAGCGACTTGAGTGGTCGAGTAATCACTCAGAATAATTGCAATGAAATTCAACCCGACAGGCGAAGCCATGAAGTTTCAAACGAAAGCAAAAATTGCAGCCAACAAATGACGCCATCAAGCCCGGAATACTCTATATCGAAATGTATGTTACGTAAGCACAAGCCAAACCGCAAACCTCGAACGCCTTTTCGCACTGAGCAACTTATGGCCCTCGAGAACAAGTTTCAGGAAAAGCAATACTTGTCGATCGCAGAACGAGCCGAGTTTTCAGCGTCACTTTCACTTTCAGAAACTCAGGTCAAAATTTGGTTTCAAAATCGTCGAGCGAAAGCAAAAAGATTACACGAGGCGGAATTCGAAAAAGTGAAGCTTGCCGCCGCTGCAGCTGCATATTCAAACCTGTTGCATGGATCAACAAGCAAGACTCATCCAATTTATCCCCCTAACATTCTTCACCGGGACTACCCAGCTGTCTCTCCTGGAATCCAAAACAACGTTGGGTTAAACCTGTCGGGTGAAATGACACCGACACAAAGGCCAAACGTGGGTGCCTTTGGCTCGTACGCATTTGTGCAATCTTCAAAACCTTCCACAATTTCCTATTTTCCTTCTGAAACGTAG**TTTTGATGCAGTACTAATATTTTTCGATATTTGTGATCGCCAAATAAACTTTTTGTTTTAAAACTTAA

>*Phmamm.Tox*_cDNA

GGCGCATGTAAATTGGAAAGTCAGACATTGTAACTCTATCCAACCTACTAAACAATCTATGTA**ATGAATGACGGTATTGAAAACTTCATGGGTTACTTAGATCAGCCTTGTGAATATTCTTTTGATGCTCCGGTGCAGAATCTGCCAACTCAAAAACAGTCTGGTTTGGATCAGAGAACTGGATACAATCGATCCAACTTTGGGAATATCCCGGCACGTTATCACCCATATAGCAGTGTTCCACGATATCAACAAACTAGTGGCAACTACCAATGTCCAACTAACTGCTACGCAGTGCAGTCAACTTATTCATTGACCGAACCGTTAAGCCACGACAGATTTAGAAACACCAGCTTTACGATGGACAATACCTCGTTAGAGCCAAACTGTAGTTTTGGAAATTCTAACAACAACAATAACAGTGTTATAGGCGGATGCAGCTACAGTATGGGGACAGTACCGGTGCCAAGCAGTTTGTATGGACAGGTCGAAACACAACATTCATCAGAAAGTTTGTACAACCAAAGCCACCCAGTAGTGTCATCAGCACTCTTATCCAATAGAAAAGAATATTCTGTGAATCAAAGGTTTTCAAACTACCCCACCTTCACTTCCAACAGGTCAAGATTATTTCAAACAATGGAAATTGATTCTGCTTCAAGTCCTGAGCAGGATTATTCAGAGGTTGATGCTGGTGGTAAAAAGAGAGGACCCAAAAAAAGGAAGAAAAAGGGTGCAAACGAACCACAGAAACCAGTTTCTGCCTATGCCTTATTTTTCAGAGACACCCAAGCTGCTATAAAGGCTGAAAATCCAAACGCTACATTCGGAGAGATTTCAAAGATTGTTGCTTCAATGTGGGATAGCTTAAGTGAAGAAGCAAAGCAGGTTTACAAACAAAAAACGGAATCAGCCAAACGAGATTACCTGAAGCAGCTTGCTGCATACAGAGCAAACTTAGTTTCGAGGGGTGGTTTGGATGCTGATGAAGATGAAAGTCAACCTTTATCTTTGCTCAAGATAAAAATGTCAGACAGTAATCATTCTGTATTGCCACCATTACCAAAATTACAGATGGCTCCCAACAGTAGCAAGAAAGACTGGTCATCTTCATTACCCAATGTTCCAGAAACTAACCTATCATCTATACCTGATGATATGCAGTTGGGGGAGGGCAGGGCACCCTCTCCATCCCTTGTTTCTGTTTCGTTTAGTGCACCCAATGGCAATTCAACTCAAACAGTACAAGTGTCACCAGGTTCACAGGCCCTTGTACCACCTCCAATACAGCTAAGGAATATTGTTCCCAAAGCTAACTCTCCTGTTGCTTTTTTACCAAGTAAACCAGGACAAGTAATAAAAGTTTTGCCAGCTAGCCAGGCTGCATCAATTGCCAACTCCCAGTCGGGGAGAACTCGAATAATTAAGATGGCTGATATGCTGCGACCTGTTGTTTCCCAGGCATCAGGTTCTGAGGTATGTTCTCCAACCCAAAGTGTTCAGCCCATTCTACAAATGTTGGCACAAGAAAATGAAGTGGAACAATCTAATTTGTCAGCATCGAGTGAACGAATTCATTTTCAACCAAACAATGCCTTAAGTAGAATAGAATGTATTGATTTGACTAACACCCCAGCACCAATATTGGAGAGTCAGGAATTTGAGCCTGTAACCAACAAGTTTGAGGATGCTACAGTTCAATACTGCATTAGGGAGGGTTGCAATAATGTTGCTGATGCAGATCCTCATTGGAATAAAGAATATTGTTCCAACGAGTGTGTTGTAAACCATTGCAGAGATGTGTTTGATGCTTGGACATCTGCTCGTAAAGTCAGTGCATCGGTCAATTGA**ATACAATGGAAACAACCTGGTGGAAAAATTATTGTTGAACAATTTGCAAAGCTTCATGAAAAACATGTGAAACAGGAAGAAAGTGGATTACTAAAAGTTGCTAATGCTTAGTTTGCTGTTTTAATTGAATTCCACA

>Asment.Msx-up

GAATGATTTTGGAACTGGCAGCACAAGTTTGGGAAGGCGAGTTTTATCCTGGTGTTAGCAACACAACTTCACATGAGTATTCAACACGTTACTTACAATAAAACCATAAAAAGTGGTCAATTCGGAAATTTTAAGATCGCAACCTTGGCAAAAGAAATATAGCCTTTGCATGCTTTGTTCCCATGTAGAAACTATCAAAGCAAACTTTGAACAATGACATCCTTGAATTGTGTTCTGAGTTTGAAGTCGTCTTTTTAGACGATGATGGAGAAAGTAATAGCCTAACTATAATTGTCCACCCTTAGAACGCTGAACCCCTCGTGTAAATTGAAGAGAACATGCGGAAATTCTAAATTGTGGATAGGCATTTTTTACATCAATTGCCAGATTATGGTGATTGCAGAAGATAAACAGCAAAAGACATTACCCCCTGCGTTGCAAACTTCGTTCATCAGCGACAATAGGTAATAAATTTTCGCAGTGCGTTTGATCTTCTTCCGAACGCAAATCTTGGCGTCGATAAACGCATAAAGTGTGTCTGATGTCTTTACTTTGTCACGTGTCTAGCACCCTATTCGGAATTTATGACCCATATTCCATAAGTGGTCAGATAACGGGCAGTTTGGCGAGTTTTGAATAACTTTAAATACGTCGAGCAAACTGTTATTAGGTTGTCATATGTAAAATATACCTCAAGTACGATATTTTGCTAAGAAACCGTTATTTTTACGTTACAATCGCAACATACATATTTTTATTTTCAGTTCCTAATTTGGATCCGTCCTATACGGAAATTAACAATACGCATCTTGACGCCATATTTCTCTGGATATAATAGTTAGATTCAGCAGTTGTTTAATGCAACTTGTTGTGTAATGGTCACATATTATCAGTGTTACCTTTCCTTTAATGAACACACAATCCCACATTATTTTAAAAACACATTTGTTTACTTGCCAGACAAGTGGGATTTAAACGATGCAAGTGTTTTCGTAGCATGTGCATTTTACAAACCAGTTTGTTTTGTCAATAATTACAAAGGTTTTGCAACACATAAGACGCCATAACAAAACGTCCCCATCCTAATGCTAGCCCATATTGATTGCACACTGTTTATTAGTGTTGATTAAACTGTAAACAGACATCATGAAACCGATAATGGCTCATTATTTTTGAATAACAATAGTTTGACTTCGTTAATCCCGTTTTCCCTCCAGACCAGGATCGCATTATAAGTCGATTGCGCTAGCCACGGCACTTAACGGGACAAAATGGCTGCAAGTCCCTTTCGGAATTGACATCCAGCCAGGGAGATCGCGGACGCCCAGCAGATTTAATTGACAACGTAAATCTCCATCATTGTGGCGGCGAGCCCAATCCGGACGTCTTGATGAAAAGGGGTGCCGCAGAACGAACGACCGCCGATAAGTGTTGCAAACACGACTTAAACAAACAAGAGAAAGATGCGTGGGGACACCGCTGACAGACGAACCGAAGTGACG

>*Moappe.Msx*_cDNA

GAAAGATTTTAAAAGGCAACTTCTCTTGTTTGAAAATTCATAACTTTGTTTGCATAACAACGTGACAGATCACATTAAATATAATAAAGAAT**ATGAGCCCATTAAAAGTAACGAATGATATGAACGCAAAGGATAGCAAAACAGAGTCAAAGAATTCGCATTTTTCAAACTATGGTAAAATGGAAACTTCGCCAAAAGAATCAACTTCGTCAAGTCATCCAAAATCATCAAGTCCGAAACCCAAGCCCAAGTTGTCGTTCAGCATACAATCTATTATGCAGGGAGATTACGGTAAACCGAAAAAGCAGCAGCTTGCAACATATTATTCCCCAACGAATGTACACGCTGGTTTAATTTTACCCAATGAAATGTCGTCTTATGTTTACGGGCGCCCAAGTTATTTCAATGGATTTGGCGATGGTCACAAAAAAAGTTTGGGCAGCCAAGTGATTCCTAGTAGATTGTCCCCTGTAGAAGCTCATAATGATGATAGCAATTCTGCAAGTTCTCCTAAAAGTGCCTCCTGGCCCTCATCTCCTAATTCGTCCATTGATGACATAAAAGAAGGAGGACTTCCCATGAAATCTAGCGAAGGATCAAATCAGGATATAAGTAAAAGCCCAGAGACTACAACCAGTGTCGTTGTCAACAACTGCCACCTGCGAAAGCACAAGGCCAATCGGAAGCCAAGGACGCCATTTACCACTCATCAACTATTGTCATTAGAAAGAAAATTTAGCGAAAAGCAATATTTATCAATCGCAGAACGAGCACAATTTTCAGCAAGTTTATCCCTCACTGAAACACAGGTCAAAATTTGGTTCCAAAATCGACGCGCCAAATCCAAAAGAATGCAAGAAGCACAAGTAGAACAAGTCAAACTGGCCGCTGCAGCCGCATGTGTGGGAGTTAGGGCTCCACCTCCAATGGCATCATGCTACCCCAACATCCTGTCCCACGCACTATACGCTGCACAGCAAAGAACTGTTGGAGGACACTTTCCAACAAGGCCATTTCATCATGACTTTCAACATGCTTTAATGAAAAATGTTCAACATCCACCACAAGTCAAAGTGGAAGCAATGTCTTCCCCAAAATGCCATCAAGGCCAAACGTTCCCCCGGACTTCCCCAATGCAAATTTCCCCCAATCACATACCTGTTGTTTCACAAGCAAGTATTTCCCAACATAGCACCATTCCCCTCAGACCTGCGTTGAGGTTTGATGGATATATGCATTCATCTCCATTAATGTGA**AGTCTACATATTGTATGTTAACTTCTTTTATATTTATTTATTTATTGTTTTGAGTAGTTTTTTTGTTTCTGCGGCAATATTTTTTGCAATAAACCATTTCAAAATCCT

>*Moappe.Ascl.bα*_cDNA

CTTACGACGTCAAACGTGAATCAAATCGTAAGTTTATCGACGATTGACGACGCCGAATCAAAACAATAACAATAGAAAGGATGATTCCGACATAAATAAAAGCAGAAGAATAACGCAAATTTTAACATTATTTTGTGTCGTAGGTGGATATAAACAGTAGTTCAATCGTAAATATTGTGTAATATATTCCAACCGCTGAT**ATGCCTTCTACTACAACTTCCGTGGAGCCAAGGAAGCGGGCAAGCGTGGAACGGCGAAACGAGAGGGAAAGACGCAGAATAAAAAATGTAAACAACGCGTTTGATGAACTCCGGCAACGTGTACCTTCGGGAAGTTGCAGCAAAAGAAAAATTAGCAAGGTTGACACGTTGCAATCGGCAATTGAATATATAAAAGTATTGGAAAATTTAGTGAAAAACCGCAGGCGCAAAGCGTCGGTTGATTGCAACAACAATCAGAGTAATTTGGCGGAAAATAACAGCAACAGCTTAACTGTGAGCGCCTCAGAAGGCATGTGTATTGACAACAACTCTAACCCGATTAACCGCTCACAAATCCATGGAGCAGAAACAAACAGTCGAGCGAATGACGACTCCATTTTCCCCACAAGCTCGATTGGAATTCAACGGAACGGCGAAAACGAAGCTGTCTTATCGCCGCACGACGGTTATATCTCTGATTACGATGGAAACGACAGTGGATTTTCCGACAACTCGCAACGAGAATCGTCAGAATATTTTGGCTCATCCCAAAACTCGCCGCTTTTCTTTTCCGACACACCCCAGGAAAATTGCCATCAAGGTTTTTTAAAAGAACTTGAGCAGCCAATCTCACCCAGACATTTAGATGATATGCTAAATGGGGTTACAGTTGAAGATGTCGAGATTTTGTGTCCATCAAACCTTTACAATAACAATCAGTGTTTCGGCCCACAAATGTCTGCATACACTGCTATTCAACCAAAACCCATTCTATCACCTTTAGCGTCGTCATATCAGTCATTGTTCGGGACACTACAAAACTGA**ACAACATAATTACTGCCAAACAATACTCGGTTCAAGCATTGCCCTGACATTCGTTGCTATATTTTTATGCAATTTCAACCTTTGTACACCTATTTCAAGTTTCAATCATCGCCACGTTTCAAAGCTGCCAAAATTTTCCGATTTGTGCC

>*Moappe.Ascl.bβ*_cDNA

AATCGATATATTCTCTATTAAAAGGTAACATATTAAAATTCGAAGCAACAAA**ATGAAAGTAGAATATGGAACAGATTTTTTAACAATGAATGCTGAAAAACAAGGCAGTGGAAAAATAATGGCTGAACCAAGGAAACGAGCCAGTGTGGAGCGCCGAAATGAGCGAGAAAGACGAAGGATTAAGAACGTTAACAACGCCTTTGACGAACTCCGTCATCGTGTGCCATCGGGAACCTCGAACAAGAGGAAAATAAGCAAGGTTGACACACTGCAATCGGCTATCGAGTATATTAAAGTATTGGAGGAGATGATCCAACAGAGAAAGGAGGCAGCTCTGAAGCAACCACAAGAACAAACCGTCGATAAAATAACAAAATTAGAGAGTGAGTATACAAATGACAGCGGATATGGCGAAGAGAACGAAAGCTGTGAAAGAATCTGTTTCGAACAAAACGGCTTTGAACAACCGGGCAAATACTTCCAAGAATCTTTGAACGAAGTGTCCGTCGACGATGTTGTAAACATTTTGTCGCCAATTGAGTCTAAACTCTCGCCAAATGTTTTTGCCCCGCCGTCCCCCATTTTTCCCATCAACAGATACCAACACCAGATGTCGCCTTTGCCAATAACTCCGCCCCCACCGCAGACTTCTACTCCAGTTTTTAACCACACTGTATTTGCTTTCCCCTCTGTACCTTCCCAACATATGCCAAATTTACAATATCTAAATTTTAAGCAACAATAA**TTGTCTTTTGATTTTGCCAAATTTTTATGCCAATTTTTATAACGAAATAAATTTATATATTTATCTAAAAAAAAAA

>*Moappe.Klf1/2/4*_cDNA

CTACTTTATTAGCTGTCGCTTACTCATAATATTTTAAGAAATTTATCACCG**ATGTTTAATCTGTGTTATTTTCAAAGGTTGCTCTACTTTAATTCAAATATAATTTATCTTACAATGAGCGTAGAAGTATTTCAGTTAACAATGGCAACTGACACGAACAACTTAACTGAAGTTTTTGACAAAGGTTCTTTAAGCGACTGGGATGAAGCAATTTGGGACAAAGTGGATGAAGCTCGCCTTGAATCGATGCTGTATAGCGATCAATCCTCTTCCACAGGAGGACTGAGCCCTGAAAGCCGGGACTCTGCAAACTCGTCACCGATTAACTTCAATGCTGATAATCTGATAGATGCTGCAACTGCAGATCAAGAATTTCCACAGATTGCGGAGAGCCTGGTTGCCGAGCTTCTCGAGCCAGTATCAGAATGTCAGCCTTTCAGCAACGGAGAATGTCAGAAATACATGGACTTGTCACCGCAGTTAACAAGTTCTTATTCAACTGCAAATTATTCTCCTCCGAGCTACAATGCTTGGGAATATGCTTCAACGCAATCCAGTGAGAAGAGCGGCGATGTTTCACCTGTAGTGTTGGATGGCACCACGACTTTTCAACAAAATTATCCACATTATTACAACAACGAAAACACAGACTTTATGTTTCAAAACCAGAACAATATGCAAATGAAAAACTCCCCTCCTTATTGTGCCCCTCCGTACCCAGGCACCCAGTGTTTCGGTCAACCAACATATCAGCACCAAAATCAGCCCCCGCAATACTTTTCAAACCAACAAGGCTCAATTTTTAACCAAGATAGCTCGACGAACGCTAAAGACCGCGCTGCAATGCAGCAGATAAACTGCAACATCTACACGACGCAAGTACACCAGCATACCAACGTTGTCTTCAACCCACACAATGCCGGCATGTTTACTAAAGAGGTTTCAAACCTTGAATACCAAAGTGCACAAGTTTCCCAAGCAATTGTTACCACCGCAGATGCAAAAGCCGGTGGCAAAACGAAAGGCACACGCAGTCGAAGGGTAACCCGCAGAAGAAAAGCGACGATACATGTGTGCAACTACATGAATTGTGGAAAAACGTACAGCAAGAGTTCTCATCTGAAAGCGCACATGCGCACGCATACAGGTGAAAAACCATATCTCTGCAACTGGCCTGGATGCGGGTGGCGATTTGCACGCTCCGATGAACTCACACGTCATTTCCGGAAGCATACAGGACACCGTCCTTTTAAATGTTCATTGTGTGAAAGAGCCTTCAGTCGATCTGATCATTTGGCGCTCCATATGAAACGTCACGTCCAAACCGAGGAGCGTTGA**AAAGTCCATCTTCTCGCGGAGACCCATGGGCGGCAGTACATGAAGAAGGCGAGGGATTTGATACTGATTTTAAGCTGGCTTGGTCAGTAAAATTCCGTTGGCCTGGCAACCACTAATCGCGCGAAATCGCGCGAAAATAACTGTGCAAAGTTGACGATCAACACGTTGTTAAACGAAAGAGAAAGAATATTTATTATTATGTTTTTGTTTTTGGTAAGGTTCCATATTCTGTTCCAATACCATACGCTCTGAAACAAGAGAGTTTTAATATATTGCTGAAATACATTTTATACGAAAAA

>*Moappe.Nkxtun3*_cDNA

GTTTTAGTAAT**ATGAGTAGTTTAAGTATGGGGAGAAGATTCTCTGAATATAAAGGGAACCTGGGCATTTGCAACGACATTACTGAAAGAAAACAGTTTGCTTGTTCTGCATTTTCCGAGTCGAGTTCTCAAAATCAGGCAAAAATCATGTCAGACGACCATAAGAAATCGCGGGAAAGATTTTCGCGCGAAGATATAAGTCAAAAAATGGAGTGTCAATCCTCGCAAATCATGAAAAATTTTTGCTCTTTTCCAAGTTCAAAATACGAACCACAGATGGATAAAGCCGATGGGAAACTCTGGTCAATTTCGTCACAAATTAATAGCCATGAAGGCAACGTTAAAACGACTTATTCAAACGTAATGCTTTCGAAATACGGCAACTTGATGAGTCAACCCGATTGCAGTAATACTCCGACTCTCTCGTTTGCAGCGACGAAACAAAATGGCTCGCAACAAAAAGAGCTGACTGCCGATAGAAAAAATAGTGAATTGTCGCAGGACGGATCAGAACCTTGCTCAGTTGACGTTGAATCCATTGATAACTCTTCACCAAGCTTCAGCGACACGTTTGGAAGAAAGCGGAAGAACAGCGATTGTGATTTCAAAATAAACGAAACAGGGGTAAATAATATGACAAAAGAGAAGTACTTGGAGATGTCGAAAAGAGAAGATCGTGTGTCAACTCACTCCATTGCAAGAATCATCGGTGAGAGTCCTCCGCGATTGTACGACACTGCAAAATCGGATTCTGATAATGAGATCAATAATAATGAAACGAGAATGCAGAACTACAAAATGCCGAGTGACAAAATGAAAAGAAGTCACCTTTCACCTGAAATTAAAAAACGACCAAGAACAGCTTTTACACCAGAACAAATCAAACGTCTTGAAACTGAGTTTCAAAGGAATAAATACCTGTCAGTTGGAAAGCGAATGGAGCTTTCGAAAGCACTCAAGCTGACAGAGACACAGATAAAAATTTGGTTTCAAAATCGTCGAACAAAATGGAAGAGAGAATATTTAAGCGAGTGGGAGGTTTGGGCGCATCAGAACTATTACGCTATGCATGGATTTTATGGCGCTGCGGCAGCTGCCAGTGCTCTTGCTGGTGGTGTTCCACAAAACAATCTTTCACGATTACCTATTGGCAATTCTCCCATTAATAACCCGTTCTCTGGAATCCAGCCTCAATTATCCACTTCACCAAGTCTTTTGAAAAACCAACCACGACCGATGCAACTGCATATGACAGGGTCCGGCATATATACGGCAGGTGGTCCTGGTTTGCCACAAAGTTTATCACCGAGATTAATGAGCAGTTTACATTCACCTGGTATGCATGTTAATCCTATGACCGCCATGTTTTCGCAAATAACTGCCGGAGTTCAAGGGCAGTCGGAATTGTCACCAACGCGTACTCAGCAGCCGTACCTTCCCCACTTGCCTTATTACATCATGCCAAACCAAATGTACACATCGTCAATGCCCATTTATGACGGAACAAAAACCGCCGAAAACACAAACAAATTACTCGGCAGATGGTCGCCTGGCAATAACAATGACGGAAAAAATGTTTCTGCCACAATTGATTCGCCGGTTTCTATGGACTCTCCAACTACCCCGACTCATCTACCACCTCTTCCAGTGGTCACTGGTCAAGCAAGACTCCATTTACCGACCGCAATAACTTGCAACGTTGGTCAAACTTTGCCGTCTTTTACAAATTGGCCAAGAGAAGTCAATGTACAATCGGACAGTGCCTGTCGTTCATTGTACAACAAGGACAACGAACTGTTAACAATAACATAA**ATATTTTGTGACACACTGATTGCACATTTAGTTTGTTTCTTTTTTAAATTATTTACTCTTCACTGAAAGTTCAATGATGTTTTTTTTCATATTTTTGCTTTGTGATTTAAAATCGTTTCAATAAAAAAATTC

>*Moappe.Tox*_isoform1_cDNA

GGTTGTATTAAGAGCCTTATCTTTATTTAGAGAAGCAGGTACAACGGCAGTAGGCCAACTGCAGTACGAGTACGTTAGGCTGGTAGGCACTGTTTACTAACAGACTAAACTGTAAAATATTTCATCCGATTTGAAGATTAGATGGACAGAAAAACATTTGGGAAACTGAATCGATGACCTGTTGACTGAATGATGAATCAGCAAACTGAGGAGTGACCAGACTGAATCAAAACAGAAAGATTGTGTAGCGCAATCGGGTTATTGGAAAACAGACGCCTAAGGCCTATCATATTTATAAGAAGAAAGAAGGATTTCTGGGAGCAATCATCTATCAACAGACACGCATGTAAATCAGTTTGGATGTGCTCCACAATTAGATTGCAATACTTATCAACCACAGTATACAAATACAGATCATCAATTTCAAAACAATCAAGGGAATTTACAACATCATAGCACTAATTTCAAATTG**ATGTCCACTATGAATCCAAGTTCTCCTCTTGATGGAAATGGTTCAAAGAAAAAAAGGTCTCCCGAACCGAAAAAGAAAAGTACGAGGCGAAAGAAAAAGGGAGTGAATGAACCCCAGAAGCCAGTGTCAGCATATGCATTATTTTTTAGAGATACACAAGCTGCAATCAAAGCAGAAAATGCATCTGCTAGTTTTGGTGAAATATCTAAAATAGTAGCGTCAATGTGGGACACCCTAAGTGAGGAGGACAAACAGATTTATAAACAAAAGACGGAAACTGCCAAACGCGATTATTTGAAGCAATTAGCTATGTATAAAGCTAACACCATTTCCATGGGCAGTAGTTTGGATGCCGATGATGAAGATTCACAGCCACTTTCGGTTTTGAAGGAAAAGATGGCCTTTTCTGCTTCAGAAATACCGTCTCCACCCCCGTCTCCTATAATAACACCCCCACAACAAATCATAGATCTCACAGAATCATCACCTGATCGCACCCTGCCTTCGTCTCCCAAGCTACCACCTTTGCCAGGAATTAGCTTTATTAAAAATGAATCAACTGAGCAACCTGAAGAAATAGAAAATGATAACACAGATGCGGATGACTCTTTGCAAAAAGATGAAGAAGAAGAAGTAGTACCACAACCTCCACAAATTCACATTAAAAGTCGATCTCCGTCTCCGACTATCGCATACAAACAAATCATAGTGTCAAATCAAAAAGGTTGCCAAGGAACGATCACATTCATATCAAAACCAGGGACCGATCCGCCACCAACTTCTTCCACATCCAGTCCACTGAGCACACAGCAGATGAAAAGGATTGTTCTAACACCAATTTCATCCACAGGTTCTTCAGTTCCTAACACTCCATCACCTCCCCCTCCTATTCAACTTCGGAATATTGTAGCAAAGTCACCAGTGACCATAATACCTTCAAAACAAGGTCAAATTATAAAGGTTTTACCAAATGGAAAGATGGGAACCGTGACTATACCAAAGGTTTCCAATGTCAAAGGTAACATGATTCAAGTAAATATCAATAGACCTCTAAGCACTGGTACCACCATAGCATCAACATCAGTACCATTACATTCACTAACTACTAACACACCAAAATCTCCACCTGAACCCAAAATCGAAAAGAAAGAAGATGATGAGATCTTAACAACAAAAATGGATGAAACTTCGATGGATGATTCTCCTCCTCAATTAGAAGAAAAACCTCTTCCTACATTGCTTGTAAAACCTGAGAAATCACATGAACCTGTTCCAATGGAAACAAGTGAAAACATAAAACCCTGCTTACCAGCGCTGCCTAAATTAAAAAAGATTCCGAAAACAAAAATTGAACAAAACACTGTGCCAATTAAAAGCGAAGTTGTGGAAGAACCTAAACCATTGGAAACAGCTTCGGCACCAGATATAAACAGACAGTCTCCAGTCACTATTATGCTAACTGCTGAGCCAGGGAAAACACATTCACAAAAAAGAAAACTTGAAAGGGGGTCAAGTAGCAGTTCAAGTTCACACACAGCTGTAAGGCAATGCATAAGGAGAAATTGTCAGAAAAGTGCTGTTAGTAATCCTGAATGGGACAATGATTATTGTGGATATGAATGCGTTGCAAAGCATTGCAAGGATGTTATGAATGCTTGGATAAGTGCTAGACAAGTCACCGCATCTCTTGGGTGA**AGAAAGTTGAAGTGTTGCGAAATAGCTACACAACTTCTCCCAAGTTCTTTTGTAGCACATGGAAGTGATGGTGCTATATATTTTGGAAGTTTTGAGGACAACTTTTGTTTTACCTTCTAAGCAATTCTATGAATTGTATTTTTGTTGAATTTACATATACCAACCATGAATTAATTTTCACACACTAGTTTTTGCAAACTTGTATTCAATTGTTTTATAAGAATTAAAAAGGA

>*Moappe.Tox*_isoform2_cDNA

TTTATTTGATGAATTGCTTTTTTTTTCGAATATTGTATAAGTTTATTTGTACTATTTTTTAAATTTATTTATTTTTAAAAGTGAATGGTTCACTCGTGTCGTTTTAAATGTCCCCGATGTTCCGAAATAATTAGTTGAAATGTATCTTTATTATTTAAATAAACTAATAATACAGAACAAAGAGTTCCGCGATAATTTCTTCATTGACCATCTGCTGCCATAAGTGCGCACTTTTTCCTTTTTGGCGTTTTTCTTTTCGTCAACGCGAGCGACTTTCTAACGAGGATTTTTTAAGCAAATTGCTTGACACAGTCTGCATTTATAAACATTATCTTCGCAGTAGTAAGTAGCAGACTACAGATATAGTGAGAAATTTTCTAGAGGAATAATTTTATTGAGATATTAAGAGAATTCAACTTTTCATT**ATGTTACAAATGATGTTTGAAGACCTGCCTCAGTTGGGCGAAGGAGGAAATCTGAGTCAGTTGCTAGACAACGACAATGCGCTGGATATCGCGACAACGTTTTCAGTTACCATGGCACAACAGTTCGAAAACGATGTTACTCCAACAAATTTAGAAGGAATTGGATACCAGCAAAATTATCCAGGCAACTGCGTGTACACGAGGGCTGACTTTCGACCGCATAATACAGCAAGATACCAGCCGTATTCCACACAACAGCGTTATGACCCGCAGATGAAACAGCAGCTTATGTACCCCAACTACATGATATCGTCTCCAAGGTGTTCAGCAATTACACAACCAACGTACACGGATGGATCATTAAACCATTCCCTGGCTTTTTCTGTCAACGAAAACTCAAATCAACAATGGATAGATCAAAATCCATCCAATTCATTCCACGATCAAAATAATAGAAACGTGAATAACAATAATTCGGTCGTATATGATAGTCCTTACCAGAGCAATCATCTATCAACAGACACGCATGTAAATCAGTTTGGATGTGCTCCACAATTAGATTGCAATACTTATCAACCACAGTATACAAATACAGATCATCAATTTCAAAACAATCAAGGGAATTTACAACATCATAGCACTAATTTCAAATTGATGTCCACTATGAATCCAAGTTCTCCTCTTGATGGAAATGGTTCAAAGAAAAAAAGGTCTCCCGAACCGAAAAAGAAAAGTACGAGGCGAAAGAAAAAGGGAGTGAATGAACCCCAGAAGCCAGTGTCAGCATATGCATTATTTTTTAGAGATACACAAGCTGCAATCAAAGCAGAAAATGCATCTGCTAGTTTTGGTGAAATATCTAAAATAGTAGCGTCAATGTGGGACACCCTAAGTGAGGAGGACAAACAGATTTATAAACAAAAGACGGAAACTGCCAAACGCGATTATTTGAAGCAATTAGCTATGTATAAAGCTAACACCATTTCCATGGGCAGTAGTTTGGATGCCGATGATGAAGATTCACAGCCACTTTCGGTTTTGAAGGAAAAGATGGCCTTTTCTGCTTCAGAAATACCGTCTCCACCCCCGTCTCCTATAATAACACCCCCACAACAAATCATAGATCTCACAGAATCATCACCTGATCGCACCCTGCCTTCGTCTCCCAAGCTACCACCTTTGCCAGGAATTAGCTTTATTAAAAATGAATCAACTGAGCAACCTGAAGAAATAGAAAATGATAACACAGATGCGGATGACTCTTTGCAAAAAGATGAAGAAGAAGAAGTAGTACCACAACCTCCACAAATTCACATTAAAAGTCGATCTCCGTCTCCGACTATCGCATACAAACAAATCATAGTGTCAAATCAAAAAGGTTGCCAAGGAACGATCACATTCATATCAAAACCAGGGACCGATCCGCCACCAACTTCTTCCACATCCAGTCCACTGAGCACACAGCAGATGAAAAGGATTGTTCTAACACCAATTTCATCCACAGGTTCTTCAGTTCCTAACACTCCATCACCTCCCCCTCCTATTCAACTTCGGAATATTGTAGCAAAGTCACCAGTGACCATAATACCTTCAAAACAAGGTCAAATTATAAAGGTTTTACCAAATGGAAAGATGGGAACCGTGACTATACCAAAGGTTTCCAATGTCAAAGGTAACATGATTCAAGTAAATATCAATAGACCTCTAAGCACTGGTACCACCATAGCATCAACATCAGTACCATTACATTCACTAACTACTAACACACCAAAATCTCCACCTGAACCCAAAATCGAAAAGAAAGAAGATGATGAGATCTTAACAACAAAAATGGATGAAACTTCGATGGATGATTCTCCTCCTCAATTAGAAGAAAAACCTCTTCCTACATTGCTTGTAAAACCTGAGAAATCACATGAACCTGTTCCAATGGAAACAAGTGAAAACATAAAACCCTGCTTACCAGCGCTGCCTAAATTAAAAAAGATTCCGAAAACAAAAATTGAACAAAACACTGTGCCAATTAAAAGCGAAGTTGTGGAAGAACCTAAACCATTGGAAACAGCTTCGGCACCAGATATAAACAGACAGTCTCCAGTCACTATTATGCTAACTGCTGAGCCAGGGAAAACACATTCACAAAAAAGAAAACTTGAAAGGGGGTCAAGTAGCAGTTCAAGTTCACACACAGCTGTAAGGCAATGCATAAGGAGAAATTGTCAGAAAAGTGCTGTTAGTAATCCTGAATGGGACAATGATTATTGTGGATATGAATGCGTTGCAAAGCATTGCAAGGATGTTATGAATGCTTGGATAAGTGCTAGACAAGTCACCGCATCTCTTGGGTGA**AGAAAGTTGAAGTGTTGCGAAATAGCTACACAACTTCTCCCAAGTTCTTTTGTAGCACATGGAAGTGATGGTGCTATATATTTTGGAAGTTTTGAGGACAACTTTTGTTTTACCTTCTAAGCAATTCTATGAATTGTATTTTTGTTGAATTTACATATACCAACCATGAATTAATTTTCACACACTAGTTTTTGCAAACTTGTATTCAATTGTTTTATAAGAATTAAAAAGGA

>*Moappe.Dlx.c*_cDNA

TGGTTGTATTAAGTTGATTTGGATTTAGTATATAGTTATAAAATAAGTGAAACAGAAAACAGGCACTTTTAAGAGAAGCCTAACAACAGTCTGATATTGTTT**ATGGACATGGTTATGCACACAGATAGCCAAATGAGCGCGTATGGATACAGCTACGAACCAGCTGCTGCCGCAGCCGCATCTCAATACTTTGGAAGTCCGAGAGGCGCTTCTGGGTTTGGAAATCCGGCTTATGGAGCTCCCGGTGGCACTTACGCAACATCACACTATCCATCTAGTCAAGTTGGTGTACAAGGACCAGTCTCAGTATCCACTGGGGGGAGCCCAGTGAGCCATGGCCTACATGGCGGATATTCGACAGCGCCTCCACCGACACTTGGTTCGACTGCTGCCACTGGAGGATTTCATGGTTTATCTGCATATGGTGCCACCGGTCACTCAACTTATGGTTTAATGAATCAATATCCAGGCGATCCGATTGGCATCCATCAAGATACATCACACCTTACTGGCGAAGTGACGATAAAAGGAAAGAAAAAGAAGATGAGAAAACCAAGAACTATTTATTCCAGTCTTCAGCTTCAAGCTCTCAATAGAAGATTCCAACAGACGCAGTATCTTGCGCTTCCAGAAAGAGCGGAGTTGGCTGCCTCTCTCGGCCTCACTCAAACACAGGTTAAAATTTGGTTTCAAAACCGACGATCAAAATTCAAGAAGTTGATGAAGCAAGGACTTCAGGATAAAGACAATCCTTTAATGACATCAAATAATAATGCTAATAACAACGCCGGATCACCAAATTCAATGACATCTCCCATGATGGCATCTATGCAGAGTAGCACTGGATCAGTTACATCAATCGATTCGGTTGGAGTTAATAATACAAATGGAGTCCCTGAAAGCAACGGCAATGGACAGCAATGGAGTCCAAGATCTGCTGAAGAACATTCACCTCAAAGTGGACAAGTACCTCCTCCAGTATCCGCCTCTGGGACACCCCCTTCCCATGCTCTCACTTCTCCACCTGTAGGAAATTATGAAATGATGACAGCTGGTCATCATCCACCCCCTACTTCTATAATGAAGCATGAAATGTCCCCTCCCTTACCAACAAACGGTTCTTCTACACCATCGGCAATGGTACCATATTCCGTCAGTGGAATGCATATGCCAGGAATGATACCTCAAGATAATCCTTATATGACAGGTGTGCCATTGCACCAAGGTTCTCTTCCAACGACGCCTATAACAGCTCAACCTCCACACCAGGCTATGATGTCATCATCATTCTGGTATGCAGGGGCTGAAGATGGTAGTGTTCCAGCTCACCCTGTAGCGCACGCAATGCCCCAACCACCGATGAGGAGTCCAACGTACTTAAAGTGA**TGATTGATGCCATTATTAACCACTGACTGGAATCATTCCAGCGCACCGGTACGACTCTCAATTTAGCACAAGTTCATATTTGCACCGAACCAATATTATCAAGACCATGTGTATGGGGCCAATGCATGTTCACTGTGAGCAGATATAGGGCTGCTGATAAATGATCACGTGACAATACTGTGGGTTGTTGCATTCATCATTGTGCGTTCCATTGTTACATAATAACTAATAGGCATTATTATATCATCAGGGACGGCAGTTGTTATTAATATTATAATTTCGTATTTTTGTACAAACTGGCTTCTGTTGGAGAAAGAAGCAAAAGATTATTATGTGCGCGCTAGACCGCGCTCTCCAAACTCTTCATATGTACATAAACATGGATACTTCATAGCTGGTTGCACTATGAACTACCGCCTTCCCACCAAATCGCTCACATCACTATATTGCCATAAACAATTTCTATTTCAAATTTCCAGAAAATTTTGGATTTTTTTAATAAAAATTATATTAAATATTTTATTTTTTCGTTGGATACTTTTTTTTTTGGAAAACTGGAGCAGGCCTGGCTATTTCACTCATTTAGTTTCTTATAATATTATTGC

>*Moappe.Dlk*_cDNA

ATTTGGTTGTATTAAGATATTATTTCTATTGGAAAGTAAATACGAATAGGGATTATATTGTATCTTGAAGACAAAACTTGTGCCGCCAAATCTATTGTGACTTAAGAAAAC**ATGTCGAGAAGCACCAGTTGTCTCGTCATCGTGTTGTTGTATCACATGGGAAATGTAGCAGATGCTGATGCTGAGTTAATCCCCTGCCCGAGCAGATGTAACTTGAGGCAAGGATTTTGTCACGAAGATGGGTCATGCCGATGTTATCCGGGCTGGCAAGGGGACAAATGTGAAGAATGCACACTGGCACCTGGTTGCAAGCAAGGCACGTGTCACCAACCGTGGCAGTGCATCTGTGAAAAGGGTTGGGGTGGGCGAAGGTGCGACAAAGATTTGAAATATTGTGAACGAAACAAACCCTGCAAGAATGGAGCCACTTGTATCAACTCCGATGGTACATATCTATGTGTATGTCCTACAGGATTCTATGGAAAAAACTGTGATCAAAAATTGTCCGAAAAAGAAACCACCACAGCACCTAGTGTTGAAAATTTAAACGACGAAAGATGCAGCTATTCTGGCAAATGCTTGATAGAAGATGGACCATATAAATGCGAAAAATGCCAATGTAATGCAGGATACGCTGGTGAACTCTGTGAGAGCAACGTCGACGAATGCACGTTGAGACCCTGTGCAAATGGAGGCATATGTCACGACGAAACAAATTATTTTTACTGTGAATGCCTTTCTGGATTCACTGGCAGATTCTGTTCTGATGATATTGACGAGTGCCGTGTGAAGAGAGATCCATGCAGCCGAAATGGCAAATGTAGCAACACTTTTGGAAGTTACAATTGTGAGTGCAATAAAGGATTTACTGGTAAAAAGTGCGAGAAGAAAATAGTTTCTGATTATCTGTATGAATCAACTACCATGGCGGCACTCATTAAATACGTCTCAATCACAAAAAAAACAACGAAACAAGTTCCGACGACAACCATTTCTACAACTGTTGAAACTGAAGAAATTGAATTAGTAGAAATTGATGAAGTCCCTATGAAAAATAATCCTAAAATAACTCGAATTACTCACGAGTTGCAAATTACTTCACTCAATGGGAAAATTTCGTCGAATGATAAAGAATTGCTGATCCACAACGAGAAGAACGACACATCATCTCAGGCGATGCAAGCGCTTACTTTTGTCTTTATGGCTTTAGCTCTAATTATTTTGGTAATAATTCTACTGGTTGTGTGGTCAAGGTTCATAAAAGGCGATGACACGTGCACAAAAGCAAATGAAAGTGAAAATTCCAAGTCCACCAGCAGAAGTTTTCAATCCATATCAGAATGTCCTCGAACGCCGTCGATTTTATCTGCAGAAGAGAGCAATCGATATGACCAAATAGCAACTAGTGAAACAACACAAAGTCTTTTGCAATATAATCACAACCAACCCGAACACACACAGAGGTTACTCGAACGAGATCCAAACTTAACCAACACATCCATGTGCATTTACACTCGGCAAAATCACATTTCTAACCCTCTTTCACCACCTCCACCTTACGTCGATTCCGTGCATACTCTGCCCCAGCGTACACCAATAGAAATGGCTGAATATTACGTTGACTTGCCTCCAGAAAATGAAACAAGAAGAAACCAAGGTCCATCATCGCAAAGACATTCAAAACATTTTACTAATGACGAATCTTCAACCCGTAGAATCCTTGTATAA**AAATTCCAATCATAATATTTTAACACTGTCGAATATTTACGTCATTATACTTTTATTATTGTTTTTATTTATTTTTCTTTTATAATTTTACAAATGTTTTGATTCGTCCATTAACGATGATAATTTTACAAACACTGTGCATGTAACTACAACGTCGCTTGG

>*Moappe.Celf3.a*_cDNA

TTTGGTTGTATTAAGAAATAACAACAACGAGTAGACGAGCTGTGAGATCTGAATATTTTACGTGTTGTGATTTGTGATAACATCGAACATCAAA**ATGGTTATGGCCGTGCAAGCCAATAGTGCGCAGATGCTCCAACATACCCTGCAATCCCCCCATGGGGCAGGACTCGGAGGCTGTGGTAGTTCGGCGCTGCCTGGATCCAATTTCATCGTCAACATCCCATTGTCCAATCAGAACGTAGAACCAATGTCAGGTTTTCTGCAGCCTCCAAACCCGAGCAGGATCTGTCACACTCCAATGAACATACATATGTCAGAGCTTGTCGACAAGGATGATGACGCAATAAAGCTCTTTATCGGACAGGTTCCGAAGACCTGGGACGAGAAAGATCTACGACCGATCTTCGAAATCTACGGCGAAATATACGAGCTGTCGATACTCCACGATAAATATACGGGCATGCACAAAGGATGCGCTTTTCTGACATACTGCAAGAAGAATTCCGCTCTTCAAGCACAAAACTATCTCCATGAGAAGAAAACACTCCCTGGGATGAACCACCCGATGCAGGTGAAGCCAGCTGACACTGTTAACAAAGGAGAGGATCGCAAGCTGTTTGTGGGCATGCTCGGAAAACGACAAACAGAAGAAGATGTCAAAAAACTTTTCGAACCGTATGGTCAGATTGAGGAATGCACTATTTTACGATTGCCTGATGGTCAAAGCAAAGGATGCTCATTCGTGAAGTTGGCGAATGCGGAAGACGCAAAGAACGCAATTGCAGCACTGCACGGAAGCCAGACTATGCCAGGAGCCTCGTCGAGTTTAGTCGTAAAACTCGCCGACACCGACAAAGAGCGAGCAGTTAGAAAAATGCAACAAATGGCAAACAACTACGGATTGGTCAGTCCAGTGGCTCTTCAACTCGGGACATACCCCGCCCACTCCATTGTAACTGGACCTGTACCTGCTGCAGGGTGGTCGCCAGTAGCTACTGCACTATCATCGGGTCAGTTTGGTCACATGACTACAGGGATCGGTCAGACTCCAATCGTCCAGTCAAATGGTCCTACAACCCCTGGCATTCCCAGCACTCCACAAAGCCCCGTGGCATCGATAACGGCCCTCAATTTGGTCCAACCACCGGTTGTTTCGCAGACAGGTGGATCTCTGTCCTCAGGCGTCAGTCACCAGGACCTATACTCCATCCCAACATATCCTGCGCAAACGCCCCCTGCAGTAGACATGCTTCAACATCCATCCTACGCACAACAACCATATACAGTAGTCTATGTTCCATCGCAACCATATGGGGGGAGTCAACTAATGCCACAAGTCGCAACAGGGGGTTTAACTACGAGTGGCACGCCACTACAACCCGCGTTGGCGCCCCAAACAACCACCATCATCAACACAAGTCCGACAGCACCACAAAAGGAGGGACCGGAAGGATGCAACCTCTTCATTTATCACCTTCCTCAGGAGTTTACCGACGCAGATCTTGCTAATGTGTTCCAACCGTTCGGATCGGTGATATCGGCGAAAGTTTTTATCGATCGAGCCACAAACCAGAGCAAATGTTTCGGTTTCGTCAGTTACGATAACCCACTGAGTGCGCAGACTGCGATTCAAACCATGAATGGATTCCAGATCGGAATGAAGCGTCTCAAAGTTCAGTTGAAGCGTCCTAAGGAACAGTCGAGACCTTACTAA**AAGTTGGCTGTGATGCTTGAAAACGACCAAATCCAAGAACCAACATCGTTGAATATTTAATGCCCATATAGTTCTTCATTTATTAATTTTTTCATGGTACTATTATTTTATTACTTTACTTTTTTTTTTTGCACATTTTCCCATCAAATTCACTTCGTGATACATTCCTGTATATTGAAAATCAACTAGAATTACTCCTCCCTGGTACTACATTACTAGCGTACTCTTCGTTGCTTACTGATAACAATCGCATATTGTTACAATACAACAGTGGTTTGTTTTATTTATTCACTGTGTTTCATCACAATGTTCTAATTATCAAATCGAACGTTACCTTACAACAATTTTTCATGCTTTTTTTTCTTCAAGATTTATTTTTATTCTGTGCTTGCGGCACATGTTTACAAGCTGTACAATATAACTGCTTTGTATATACCTTCGCTTATTTTAATACTACGATCATTTATTTTTATTTATCTCAATACACAAAGTTTTTAATCTACCTAGGAATAACTGCAAACTGTTTGGCCACTTTATTGTGGCACAATAGCTGTTATAATCATGTTTGTCTTGGATATTATGACATCACATGTGCAATTATAAACTTGAATTTTTCAAATTTCTTTTCTTTTGAATTTCCAAAAATTAAATTTGTGCTATTATGACATCACAACCAGTGTTTATTTCACAATCATGTTTTCATTATTAATTTTAGCTTTTACATTGTACATACATTTTCCCTTCGTTCACAATATTGCCATATCTAATGAGACTTTATTAAATGACTCCTAACGTCAATCTGTTTTTTTTTTAAAAGCCACCTTTGCTTGTTATTTTTACCCCCTAGTTTGAGACTAAATACCTATTTGATGACTAGCATTTAACTGTGGTATCCTCAGC
